# *Plasmodium* co-infection protects against chikungunya virus-induced pathologies

**DOI:** 10.1038/s41467-018-06227-9

**Published:** 2018-09-25

**Authors:** Teck-Hui Teo, Fok-Moon Lum, Khairunnisa Ghaffar, Yi-Hao Chan, Siti Naqiah Amrun, Jeslin J. L. Tan, Cheryl Y. P. Lee, Tze-Kwang Chua, Guillaume Carissimo, Wendy W. L. Lee, Carla Claser, Ravisankar Rajarethinam, Laurent Rénia, Lisa F. P. Ng

**Affiliations:** 1 Singapore Immunology Network, Agency for Science, Technology and Research (A*STAR), 8A Biomedical Grove, Immunos Building Level 4, Singapore, 138648 Singapore; 2 NUS Graduate School for Integrative Sciences and Engineering, National University of Singapore, 28 Medical Drive, Centre for Life Sciences #05-01, Singapore, 117456 Singapore; 3 Institute of Molecular and Cell Biology, Agency for Science, Technology and Research (A*STAR), 61 Biopolis Drive, Proteos, Singapore, 138673 Singapore; 4 Institute of Infection and Global Health, University of Liverpool, The Ronald Ross Building, 8 West Derby Street, Liverpool, L69 7BE UK

## Abstract

Co-infection with *Plasmodium* and chikungunya virus (CHIKV) has been reported in humans, but the impact of co-infection on pathogenesis remains unclear. Here, we show that prior exposure to *Plasmodium* suppresses CHIKV-associated pathologies in mice. Mechanistically, *Plasmodium* infection induces IFNγ, which reduces viraemia of a subsequent CHIKV infection and suppresses tissue viral load and joint inflammation. Conversely, concomitant infection with both pathogens limits the peak of joint inflammation with no effect on CHIKV viraemia. Reduced peak joint inflammation is regulated by elevated apoptosis of CD4^+^ T-cells in the lymph nodes and disrupted CXCR3-mediated CD4^+^ T-cell migration that abolishes their infiltration into the joints. Virus clearance from tissues is delayed in both infection scenarios, and is associated with a disruption of B cell affinity-maturation in the spleen that reduces CHIKV-neutralizing antibody production.

## Abstract (web summary)

Chikungunya virus (CHIKV) and *Plasmodium* co-infections have been reported in humans, but effects of the two pathogens on each other are unclear. Here, Teo et al. show in mice that *Plasmodium* infection affects CHIKV-specific T and B cell responses, leading to reduced joint inflammation.

## Introduction

Arthralgic alphaviruses are mosquito-borne pathogens that induce musculoskeletal disease accompanied by fever, rash and joint pain in infected patients^1^. Over the past two decades, the spread of arthralgic alphaviral diseases has accelerated^2^ and raised public-health concern due to epidemics of Chikungunya (CHIKV), O’nyong’nyong, Sindbis, Ross River, Barmah Forest and Mayaro viruses in humans^1^. Outbreaks of these alphaviruses are usually restricted to specific continents^3–7^. However, since the initial outbreaks on islands of the Indian Ocean in 2004, CHIKV has rapidly spread into India, Southeast Asia and tropical America and ongoing local transmission is now established in many of these affected countries^8^.

The expansion of CHIKV into areas with endemic malarial *Plasmodium* parasites in circulation increases the likelihood of co-infection between CHIKV and *Plasmodium*. High-risk areas of co-infection include Africa, India, Southeast Asia and Latin America^9,10^, and have confirmed the presence of both CHIKV and *Plasmodium* in affected patients from seroprevalence studies^11–17^. Although most co-infection reports are derived from African cohorts^11–17^, the global frequency of CHIKV and *Plasmodium* co-infection is likely under-estimated as arbovirus screening is not systematic but performed only when patients are negative for malaria infection^17^. In addition, while *Aedes* mosquitos are the principal vector for CHIKV, typical malaria vectors such as *Anopheles funestus* and *A. coustani*, collected in the field were recently shown to be infected with CHIKV^18^. This increases the likelihood of concurrent co-infection of *Plasmodium* and CHIKV via competent vectors infected with both pathogens.

The impact of *Plasmodium* and arbovirus co-infection on host susceptibility and pathological severity is largely unknown. Our previous work reported the impact of CHIKV co-infection on malaria pathogenesis in-vivo using a mouse model infected with *P. berghei*-ANKA (PbA)^19^. We found that concurrent co-infection with CHIKV led to protection against parasite-induced neuropathology, specifically preventing experimental cerebral malaria (ECM)-induced mortality by disrupting the CXCR3 chemokine–chemokine receptor network^19^.

In this present study, we explored the effects of *Plasmodium* co-infection on the severity of CHIKV infection and virus-induced arthralgia. We found that co-infection suppresses CD4 + T-cell responses to protect against severe CHIKV-induced joint pathology, while disrupted B-cell affinity maturation in the spleen delays viral resolution in the joints. This is the first study to describe *Plasmodium-*induced host immune regulation that directly alters virus clearance and CHIKV-induced joint pathology during co-infection. These findings could be applied to improve management of chikungunya disease in regions with *Plasmodium* co-endemicity.

## Results

### Co-infection prevents severe CHIKV joint inflammation

**Fig. 1:**
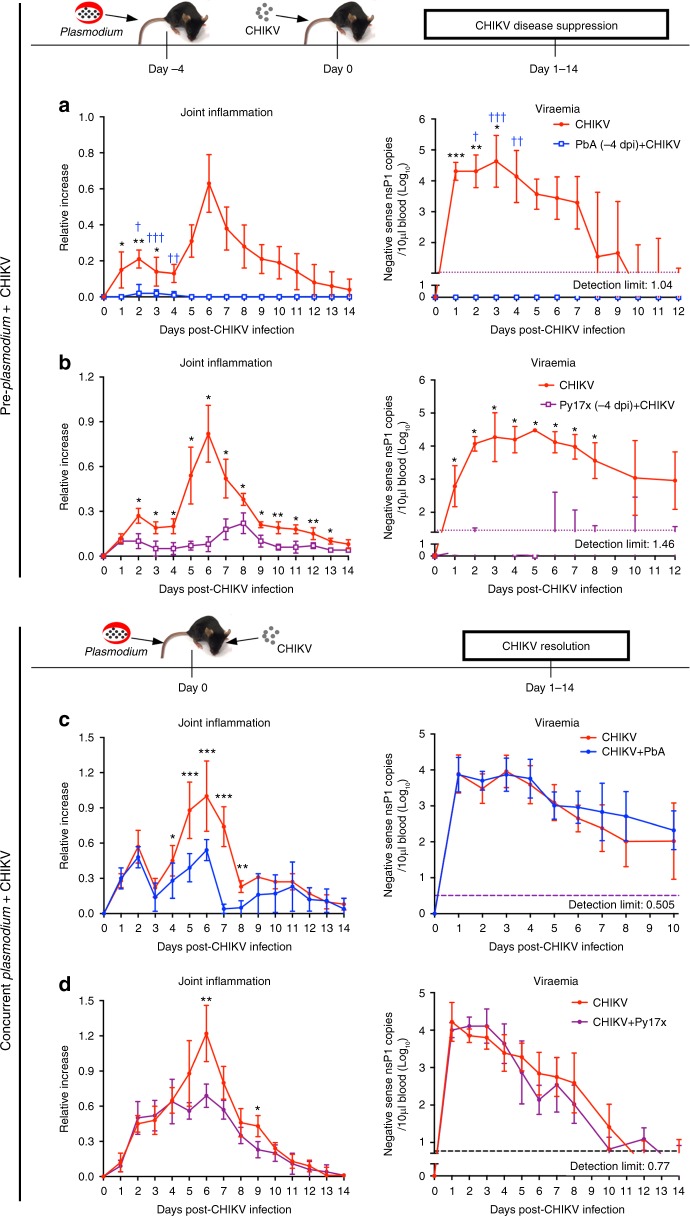
Co-infection suppresses CHIKV-induced joint swelling and viral replication. **a** Joint inflammation and viraemia of CHIKV (*n* = 7) and PbA (−4 dpi) + CHIKV (*n* = 7) groups. Each blue “†“ symbol represents one mouse that died of experimental cerebral malaria on the respective day. **b** Joint inflammation and viraemia of CHIKV (*n* = 5) and Py17x (−4 dpi) + CHIKV (*n* = 4) groups. For (**a**) and (**b**), *Plasmodium* infection was given 4 days prior to CHIKV infection, as shown in the schematic. **c** Joint inflammation and viraemia of CHIKV (*n* = 7) and CHIKV + PbA (*n* = 7) groups. **d** Joint inflammation and viraemia of CHIKV (*n* = 5) and CHIKV + Py17x (*n* = 5) groups. For (**c**) and (**d**), *Plasmodium* and CHIKV infection occurred concurrently, as shown in the schematic. All data were analyzed by Mann–Whitney two-tailed test (*P *<* 0.05, **P *<* 0.01 and ***P *<* 0.001), and are representative of two independent experiments. Data represent the means ± standard deviation. Parasitaemia were not altered by co-infection and data has been published in Teo et al.^19^. Abbreviations: CHIKV, Chikungunya virus; dpi, days post infection; PbA, *Plasmodium berghei*-ANKA; Py17x, *Plasmodium yoelii* 17×

In this study, we used the well-defined CHIKV joint-footpad mouse model where CHIKV infection alone induces measurable joint swelling that peaks at ~6 days post infection (dpi) and lasts ~ 14 dpi, with a viraemic profile of 10–12 dpi^20,21^. We also used two different species of rodent *Plasmodium*, PbA which causes fatal ECM, and Py17x which induces uncomplicated, non-lethal malaria. To understand the impact of *Plasmodium* infection on CHIKV-induced pathology, four different CHIKV co-infection scenarios were designed to reflect situations where co-infection of CHIKV and *Plasmodium* occur concurrently or sequentially^11–17^. In the first scenario, mice were pre-infected with PbA or Py17x, 4 days before CHIKV infection when mice mount acute *Plasmodium-*induced responses at the time of co-infection (Fig. 1a, b). In the second scenario, mice were concurrently co-infected with PbA or Py17x and CHIKV (Fig. 1c, d). In the third scenario, animals were infected with PbA or Py17x 4 days after CHIKV infection when mice mount acute CHIKV-induced responses at the time of co-infection (Supplementary Fig. 1a, b). Lastly, in the fourth scenario, animals were infected with CHIKV 26 days after Py17x infection, when mice had recovered from Py17x-induced malaria (Supplementary Fig. 1c).

Mice pre-infected (−4 dpi) with lethal PbA or non-lethal Py17x have abolished CHIKV-induced joint swelling and reduced or prevented viral load in the blood throughout the entire course of disease (Fig. 1a, b and Suplemenetary Fig S5). Consistent with previous findings^19^, 80% of the co-infected PbA (−4 dpi) + CHIKV mice succumbed to ECM 6–8 days after parasite infection. As such, data from the PbA (−4 dpi) + CHIKV co-infection scenario were not statistically significant from 4 dpi onwards (i.e. 8 days after parasite infection) (Fig. 1a). Concurrent CHIKV with PbA or Py17x co-infection suppressed peak joint swelling (~ 50%) with no effect observed for joint swelling or viraemia (Fig. 1c, d). No effects on joint swelling or viraemia were observed in mice infected with PbA or Py17x 4 days after CHIKV infection (Supplementary Fig. 1a, b) or when mice were infected with CHIKV after recovery from prior Py17x infection (Supplementary Fig. 1c).

Together, pre- and concurrent *Plasmodium* co-infection protects against CHIKV-induced pathology to different degrees. Importantly, the impact of co-infection on CHIKV pathology was not limited to one *Plasmodium* species. Thus, all subsequent studies mimicking concurrent *Plasmodium* and CHIKV co-infection were performed using PbA^19^. Pre-*Plasmodium* (−4 dpi) and CHIKV co-infection were performed using Py17x due to the high death rate of PbA-infected mice^19^.

### Co-infection delays CHIKV resolution in the joint

CHIKV replication persists in the joints for weeks after systemic viral load is resolved^20,22^. To understand the impact of co-infection on virus persistence in the joints, an infectious clone of CHIKV expressing a firefly luciferase marker that allowed tracking of virus dissemination and replication was used^20^.

**Fig. 2:**
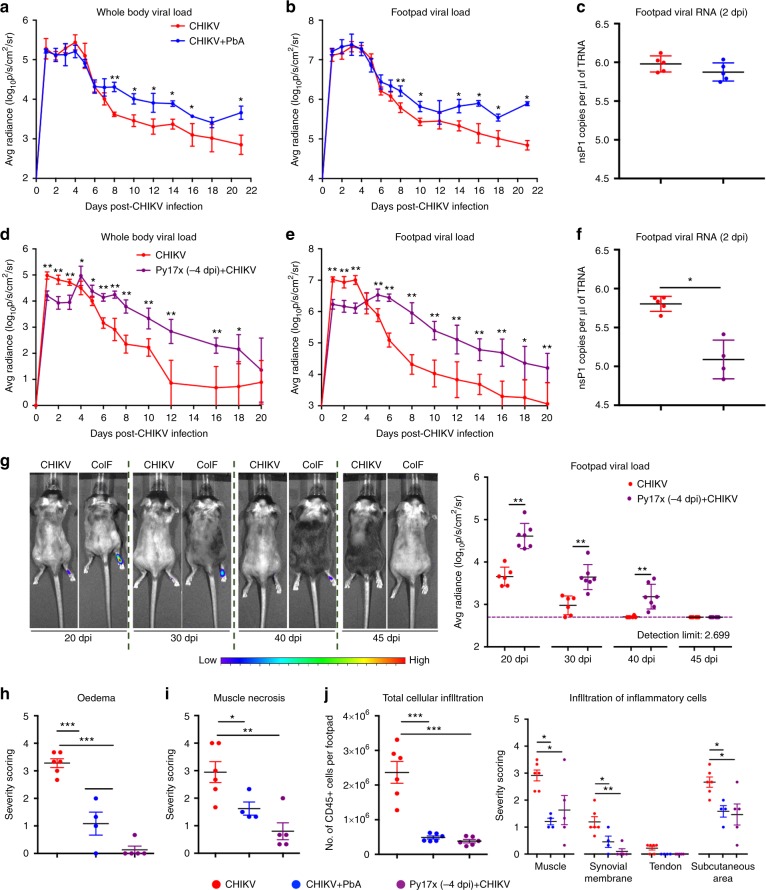
Co-infection delays virus resolution in tissues and suppress acute CHIKV-induced joint pathology. Tissue viral load in **a** the whole body and **b** the joints of CHIKV (*n* = 5) and CHIKV + PbA (*n* = 6) groups. Follow-up was terminated at 22 dpi as co-infected mice died of hyper-parasitaemia between 23 and 26 dpi. **c** Footpad viral RNA load on 2 dpi in the joints of CHIKV (*n* = 5) and CHIKV + PbA (*n* = 5) groups. Tissue viral load in **d** the whole body and **e** the joints of CHIKV (*n* = 7) and Py17x (−4 dpi) + CHIKV (*n* = 5) groups. **f** Footpad viral RNA load on 2 dpi in the joints of CHIKV (*n* = 5) and Py17x (−4 dpi) + CHIKV (*n* = 4) groups. **g** Footpad viral load of CHIKV (*n* = 6) and Py17x (−4 dpi) + CHIKV (*n* = 7) groups at 20 dpi, 30 dpi, 40 dpi and 45 dpi. Bioluminescence signal from the luciferase-tagged virus in both groups fell below the detection limit at 45 dpi. Representative pseudo-color images of bioluminescence signal at 20 dpi, 30 dpi, 40 dpi and 45 dpi are shown. Histopathological scoring of (**h**) oedema in the subcutaneous region and (**i**) muscle necrosis in the joints of CHIKV, CHIKV + PbA and Py17x (−4 dpi) + CHIKV (*n* ≥ 4 per group) at 6 dpi. **j** Total CD45 + cellular infiltration (flow cytometry) and infiltration of inflammatory cells in different regions of the joint (histological grading) of CHIKV, CHIKV + PbA and Py17x (−4 dpi) + CHIKV (*n* ≥ 4 per group) at 6 dpi. Data in **a**–**g** were analyzed by Mann-Whitney two-tailed test and **h**–**j** by one-way ANOVA with Tukey *post hoc* test (*P *<* 0.05, **P *<* 0.01 and ***P *<* 0.001). Data represent the means ± SD. Abbreviations: CHIKV, Chikungunya virus; CoIF, co-infected; dpi, days post infection; PbA, *Plasmodium berghei*-ANKA; Py17x, *Plasmodium yoelii* 17×

Interestingly, concurrent CHIKV + PbA co-infection had no effect on early acute CHIKV replication (1–7 dpi) and levels of joint footpad viral RNA (2 dpi), but resulted in higher levels of virus persistence in the inflamed joints from 8–22 dpi (Fig. 2a-c and Supplementary Movie 1) despite similar viraemia levels to CHIKV-infection alone (Fig. 1c). Similarly, resolution of disseminated virus in the tails and contra-lateral footpad was delayed by ~ 5 days in the co-infected mice (Supplementary Movie 1). Follow-up of tissue viral load beyond 22 dpi was not possible as co-infected mice succumb to PbA-induced hyperparasitaemia by 23–24 dpi^19^.

In contrast to concurrent PbA co-infection, pre-Py17x (−4 dpi) + CHIKV co-infection suppressed early CHIKV replication in the tissue from 1–3 dpi and levels of joint footpad viral RNA on 2 dpi (Fig. 2d–f and Supplementary Movie 2). Despite this early phenotype, pre-Py17x (−4 dpi) + CHIKV co-infection led to elevated tissue viral load from 5–18 dpi in the co-infected mice compared to CHIKV-only mice (Fig. 2d, e and Supplementary Movie 2). Viral load within the joints of co-infected mice remained significantly higher until 40 dpi, and virus clearance was delayed from 40 dpi to 45 dpi (Fig. 2g).

### Co-infection suppresses acute CHIKV joint pathology

Histopathological assessment was next performed on the joint tissues from CHIKV + PbA (concomitant infection) and Py17x (−4 dpi) + CHIKV infected mice at 6 dpi, when peak joint swelling was suppressed in both models (Fig. 1). Haematoxylin and eosin (H&E) staining of the joint-footpad revealed that both scenarios of co-infection reduced subcutaneous oedema and severe muscle necrosis, and prevented inflammatory cell infiltration into the muscles, synovial membrane and subcutaneous region (Fig. 2h–j and Supplementary Fig. 2). This phenomenon was also observed by the drastic reduction (4–5 fold) of CD45 + leukocyte infiltrates in the joints of co-infected mice when quantified by flow cytometry (Fig. 2j).

Joint pathology was then assessed at a late time-point to observe for increased virus persistence in the joint as a result of co-infection. Joints from pre-Py17x (−4 dpi) + CHIKV co-infected mice were harvested for histopathological assessment at 45 dpi, when tissue viral load has been resolved in the co-infected mice (Fig. 2g). At 45 dpi, the acute CHIKV-induced joint phenotypes (extensive subcutaneous oedema, cellular infiltration and muscle necrosis) were resolved (Supplementary Fig. 3a). Both CHIKV-only and pre-Py17x (−4 dpi) + CHIKV mice displayed equivalent levels of muscle fiber regeneration and synovial hyperplasia at the joint and tendon synovial lining suggesting similar states of joint regeneration despite the longer persistence of a higher viral load in the tissues of co-infected mice (Fig. 2g and Supplementary Fig. 3).

### IFNγ mediates acute CHIKV pathology in pre-Py17x co-infection

We next focused on identifying the mechanisms induced by *Plasmodium* that modulate CHIKV disease during *Plasmodium* co-infection. IFNγ is a dominant cytokine typically induced during acute *Plasmodium* infection^23^, and previous studies have shown that absence of IFNγ aggravates CHIKV viraemia in mice, suggesting a possible anti-viral role^20^. Therefore, the effects of concurrent CHIKV + PbA co-infection and pre-Py17x (−4 dpi) + CHIKV infection were investigated in IFNγ^-/-^ mice. In both WT and IFNγ^-/-^ mice, concurrent co-infection presented similar pathologies with reduced joint swelling (Supplementary Fig. 4), suggesting that IFNγ is not a mediator of pathological changes to CHIKV disease during concurrent PbA and CHIKV co-infection.

**Fig. 3:**
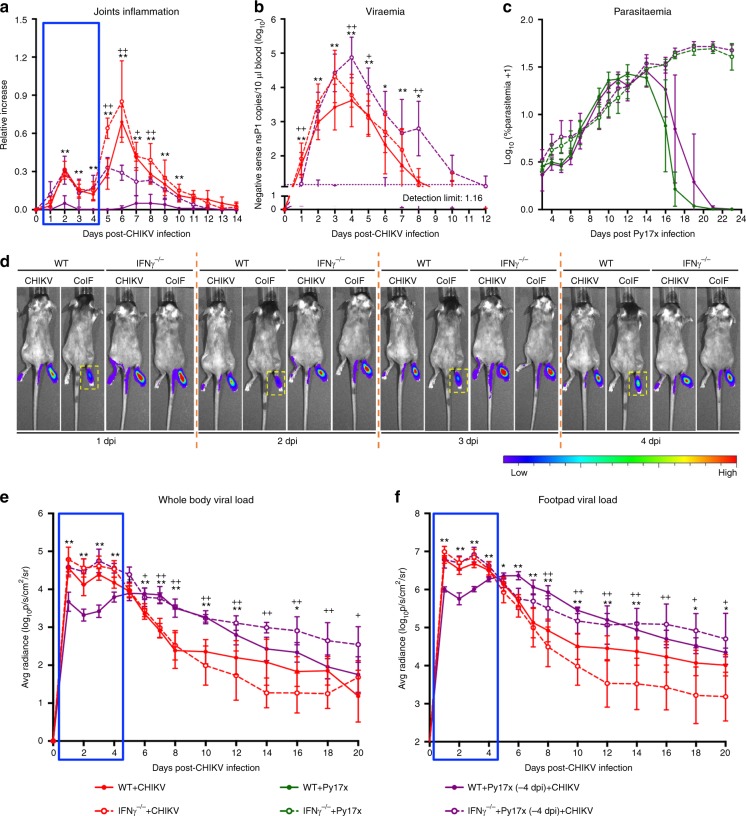
Acute IFNγ abrogates early acute CHIKV-induced joint inflammation, viremia and tissue viral load during sequential infection. Joint inflammation (**a**), viraemia (**b**) and viral load in the whole body (**e**) and footpad (**f**) of WT + CHIKV (*n* = 6), WT + Py17x (−4 dpi) + CHIKV (*n* = 6), IFNγ^-/-^ + CHIKV (*n* = 6) and IFNγ^-/-^ + Py17x (−4 dpi) + CHIKV (*n* = 5) groups. **c** Parasitaemia in WT + Py17x (*n* = 5,), WT + Py17x (−4 dpi) + CHIKV (*n* = 5,), IFNγ^-/-^ + Py17x (*n* = 4) and IFNγ^-/-^ + Py17x (−4 dpi) + CHIKV (*n* = 5) groups. Py17x or co-infected mice were euthanized at 23 days post Py17x infection as they were suffering from hyperparasitaemia and severe anemia. **d** Representative pseudo-colored images showing viral load in the tissue from 1–4 dpi. Yellow dotted box highlights acute suppression of the viral load in co-infected (CoIF) WT mice that is absence in CoIF IFNγ^-/-^ mice. Blue box in **a**, **b**, **e** and **f** denotes acute phenotypes (1–4 dpi) induced in CoIF WT mice that are ameliorated in CoIF IFNγ^-/-^ mice. Mann-Whitney two-tailed test was used to compare CoIF mice against their respective CHIKV-infected or Py17x-infected controls in the same genetic background. Comparisons between WT + CHIKV and WT + Py17x (−4 dpi) + CHIKV are represented by *P *<* 0.05 and **P *<* 0.01; comparisons between IFNγ^-/-^ + CHIKV and IFNγ^-/-^ + Py17x (−4 dpi) + CHIKV are represented by ^*+*^*P* *<* 0.05 and ^*++*^*P* *<* 0.01. Data represent the means ± SD and are representative of two independent experiments. Abbreviations: CHIKV, Chikungunya virus; dpi, days post infection; Py17x, *Plasmodium yoelii* 17× ; WT, wild type

In contrast, pre-Py17x (−4 dpi) + CHIKV co-infection in IFNγ^-/-^ mice induced joint swelling similar to WT CHIKV-infected mice at early acute infection of 1–4 dpi (Fig. 3a). Prolonged viraemia was also observed during the entire disease of 1–12 dpi (Fig. 3b). Co-infection did not alter parasitaemia when compared to single Py17x-infected mice in WT or IFNγ^-/-^ background (Fig. 3c). However, IFNγ-deficiency elevated parasitaemia on 3–6 and 16–23 days post Py17x infection (Fig. 3c). Although tissue viral load of pre-Py17x co-infected mice was similar to that in CHIKV-infected IFNγ^-/-^ mice on 1–4 dpi, levels were higher at the later phase (6–20 dpi) (Fig. 3d–f). The absence of IFNγ only partially increased joint swelling in pre-Py17x co-infected mice at later stages of disease (5–10 dpi) to a similar degree as concurrently co-infected WT mice (Fig. 1a and Fig. 3a).

To verify these effects observed in pre-Py17x co-infected IFNγ^-/-^ mice, we performed pre-Py17x co-infection of WT mice with IFNγ neutralization during the early acute phase (−4 dpi to 4 dpi) of the disease. Similarly, IFNγ neutralization restored early acute (1–4 dpi) CHIKV phenotypes (i.e joint swelling, viraemia, whole body and joint viral load) in the pre-Py17x co-infected mice (Supplementary Fig. 5).

### Co-infection limits CHIKV-neutralizing antibody production

**Fig. 4:**
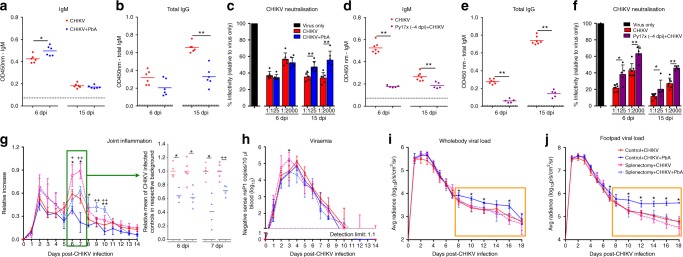
Elevated tissue viral load during is associated with suppression of CHIKV neutralizing antibodies production in the spleen. CHIKV-specific IgM (**a**) and total IgG (**b**) titer in the sera of CHIKV (*n* = 6) and CHIKV + PbA (*n* = 6) mice at 6 dpi and 15 dpi. **c** Neutralization of CHIKV infection in HEK293T cells using serum from CHIKV (*n* = 6) and CHIKV + PbA (*n* = 6) groups at 6 dpi and 15 dpi. CHIKV-specific IgM (**d**) and total IgG (**e**) titer in the sera of CHIKV (*n* = 7) and Py17x (−4 dpi) + CHIKV (*n* = 5) on 6 dpi and 15 dpi. **f** Neutralization of CHIKV infection in HEK293T cells using serum from CHIKV (*n* = 7) and Py17x (−4 dpi) + CHIKV (*n* = 5) groups at 6 dpi and 15 dpi. CHIKV-specific IgM and IgG titers were determined at 1:200 and 1:2000 respectively. Black dotted lines represents average OD reading of naïve mice for IgM and IgG. CHIKV neutralization assay was determined at 1:125 and 1:2000 dilution. Data in **a**–**f** were analyzed by Mann–Whitney two-tailed test (*P *<* 0.05 and **P *<* 0.01) and are representative of two independent experiments. **g** Joint inflammation, h, viraemia and viral load in the whole body (**i**) and footpad (**j**) of Control + CHIKV (*n* = 5), Control + CHIKV + PbA (*n* = 4), Splenectomy + CHIKV (*n* = 5) and Splenectomy + CHIKV + PbA (*n* = 5) groups. The green box denotes joint measurement during peak swelling whereby data were transformed to be expressed as relative to the mean of CHIKV infected mice in the respective background. The orange box denotes elevated tissue viral load in the control + CHIKV + PbA mice, which is prevented in the splenectomy + CHIKV + PbA mice. Data from co-infected mice were compared with their respective CHIKV infected controls by Mann-Whitney two-tailed analysis. Control + CHIKV versus Control + CHIKV + PbA comparison (*P *<* 0.05); Splenectomy + CHIKV versus Splenectomy + CHIKV + PbA (^*+*^*P* *<* 0.05 and ^*++*^*P* *<* 0.01). Data represent the means ± SD. Abbreviations: CHIKV, Chikungunya virus; dpi, days post infection; ns, not significant; OD, optical density; PbA, *Plasmodium berghei*-ANKA; Py17x, *Plasmodium yoelii* 17×

B cells and CHIKV-neutralizing antibodies are essential for virus clearance^24^. As *Plasmodium* co-infections aggravate tissue viral load only in the later stages of CHIKV infection (Fig. 2), IgM and IgG antibody titers were quantified. At 6 dpi of concurrent PbA + CHIKV co-infection, higher CHIKV-specific IgM titers were observed (Fig. 4a), while IgG titers (Fig. 4b) and neutralizing capacities (Fig. 4c) were similar to mice infected with CHIKV alone. However, lower CHIKV-specific total IgG titers was detected coupled with a reduction in CHIKV neutralization in co-infected mice at 15 dpi (Fig. 4b, c and Supplementary Fig. 6a). In the pre-Py17x (−4 dpi) + CHIKV co-infection scenario, mice showed lower levels of CHIKV-specific IgM and IgG and reduced CHIKV neutralization compared to mice infected with CHIKV alone at both 6 and 15 dpi (Fig. 4d–f and Supplementary Fig. 6b).

### Spleen dysregulation alters CHIKV antibodies production

Splenic B-cell response is disrupted during *Plasmodium* infection due to germinal center (GC) dysregulation^25–29^. To determine if disruption in the spleen is the primary reason for the suppression of CHIKV-specific antibodies in *Plasmodium* and CHIKV co-infected mice (Fig. 4a–f), concurrent PbA + CHIKV co-infection was performed in splenectomised mice. Pre-Py17x co-infection was excluded due to the impact of *Plasmodium* induced IFNγ at the point of CHIKV infection that suppressed early acute CHIKV viral load (Fig. 3).

Interestingly, peak joint swelling at 6–7 dpi was increased by splenectomy in CHIKV-infected mice with no effect on viraemia and tissue viral load (Fig. 4g–j). Concurrent co-infection reduced peak joint swelling in splenectomised mice as in non-splenectomised controls (Fig. 4g; right panel), with no effect on viraemia (Fig. 4h). However, splenectomy restored the ability of co-infected mice to resolve tissue viral load in 8–18 dpi to comparable levels as non-splenectomised CHIKV-infected mice (Fig. 4i, j). CHIKV-specific IgM, total IgG titers and CHIKV neutralizing antibodies were also similar in both splenectomised CHIKV-infected and co-infected mice (Supplementary Fig. 7a-c). Nonetheles, splenectomy also reduced parasitaemia in co-infected mice during the later phase (10–18 dpi) of the disease (Supplementary Fig. 7d). The possible indirect impact of reduced parasitaemia on the tissue viral load in the co-infected splenectomised mice remains to be determined.

### Co-infection delays germinal center B-cell generation

PbA infection impairs the GC response^26,28^ that could disrupt production of CHIKV antibodies. By adapting the flow cytometry gating strategy used in a recent study^30^, GC B cells were defined by B220^+^IgD^−^GL7^+^CD95^+^CD38^−^ surface markers. Concurrent PbA and CHIKV co-infection suppressed early generation of GC B cells in the spleen at 6 dpi (Supplementary Fig. 8b; blue dots). Interestingly, a significant increase in the B220^+^GL7^+^CD95^+^CD38^+^ B-cell subset was observed in co-infected mice at 6 dpi, which was not present in CHIKV-infection only controls (Supplementary Fig. 8b). This CD38^+^ B-cell subset was previously suggested to be either precursors of GC-independent memory B cells or GC B-cell^31^. At 15 dpi, the level of GC B cells in the spleen were similar between CHIKV-infected and co-infected mice (Supplementary Fig. 8b). However, early reduction of GC B cells at 6 dpi was associated with a decline in GC-dependent CD73^+^ memory B cells^31^ at 15 dpi in co-infected mice (Supplementary Fig. 8c).

### Co-infection limits CD4^+^ T-cell infiltration in the joints

**Fig. 5:**
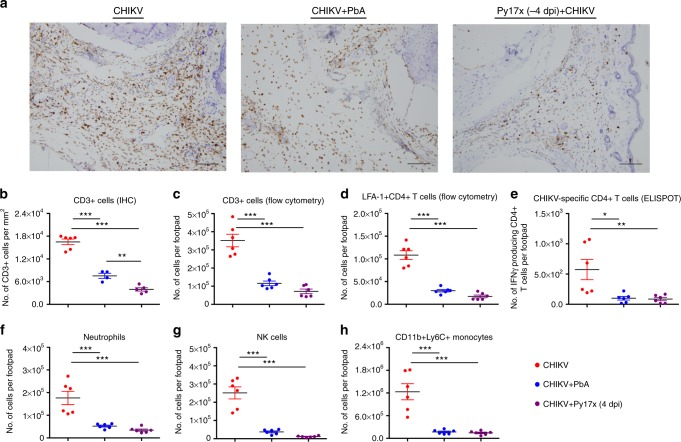
Co-infection limits pathogenic CD4 + T-cell and pro-inflammatory immune-cell infiltration into the joints. **a** Representative IHC images of CD3 staining in the joints at 6 dpi. Scale bar: 100 μm. Quantification of CD3 + cells (IHC) (**b**), CD3 + cells (flow cytometry) (**c**), activated (LFA-1 + ) CD4 + T cells (flow cytometry) (**d**) and CHIKV-specific CD4 + T cells (ELISPOT) (**e**) in the joints of CHIKV, CHIKV + PbA and Py17x (−4 dpi) + CHIKV (*n* ≥ 5 per group) at 6 dpi. Flow cytometric quantification of neutrophils (**f**), NK cells (**g**) and CD11b + Ly6C + monocytes (**h**) in the joints of CHIKV, CHIKV + PbA and Py17x (−4 dpi) + CHIKV (*n* ≥ 5 per group) at 6 dpi. Data represent the means ± SD and were analyzed by one-way ANOVA with Tukey *post hoc* test (*P *<* 0.05, **P *<* 0.01 and ***P *<* 0.001). Each data point in the dot plot represents one mouse. Data are representative of two independent experiments. Activated CD4 + T cells are defined as CD45 + /CD3 + CD4 + /LFA-1 + cells. Neutrophils and NK cells are defined as CD45 + /CD11b + /Ly6G + and CD45 + /NK1.1 + cells, respectively. CD11b + /Ly6C + monocytes were gated after gating out all T cells, neutrophils, NK cells and B cells. Abbreviations: CHIKV, Chikungunya virus; dpi, days post infection; IHC, immunohistochemistry; NK, natural killer; ns, not significant; PbA, *Plasmodium berghei*-ANKA

To understand how co-infection alters the joint infiltrating immune subsets, immune cells were profiled by immunohistochemistry (IHC) staining and flow cytometry at 6 dpi during the peak of joint swelling. Both concurrent CHIKV + PbA and pre-Py17x (−4 dpi) + CHIKV co-infections reduced CD3^+^ T-cell infiltration into the subcutaneous region of the inflamed joints compared to CHIKV-infection alone (Fig. 5a, b). Flow cytometric profiling and ELISPOT assays of isolated CD4^+^ T cells also showed that *Plasmodium* co-infection abrogates infiltration of activated (LFA-1^+^) and IFNγ-secreting CHIKV-specific CD4^+^ T cells into the joints (Fig. 5c–e). Finally, co-infection also suppressed infiltration of other immune subsets, including neutrophils, NK cells and CD11b^+^Ly6C^+^ monocytes/macrophages into the joints (Fig. 5f–h).

### Lymph node dysregulation alters CHIKV joint swelling

**Fig. 6:**
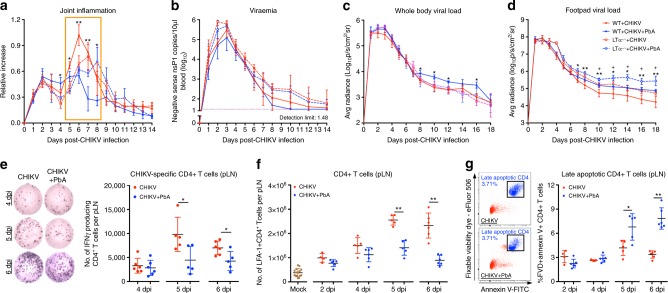
Co-infection induces early apoptosis of CD4 + T cells in the popliteal lymph node (pLN). Joint inflammation (**a**), viraemia (**b**) and viral load in the whole body (**c**) and footpad (**d**) of WT + CHIKV (*n* = 5), WT + CHIKV + PbA (*n* = 5), LTa^-/-^ + CHIKV (*n* = 4) and LTa^-/-^ + CHIKV + PbA (*n* = 4) groups. The orange box denotes suppression of joint inflammation (5–8 dpi) in co-infected WT mice compared to WT + CHIKV, which is lost in the co-infected LTa^-^^/^^-^ mice. Data from co-infected mice was compared with their respective CHIKV infected controls on the same genetic background using Mann-Whitney two-tailed analysis. Comparisons between WT + CHIKV and WT + CHIKV + PbA are represented by *P *<* 0.05 and **P *<* 0.01; comparisons between LTa^-/-^ + CHIKV and LTa^-/-^ + CHIKV + PbA are represented by ^*+*^*P* *<* 0.05. Quantification of CHIKV-specific CD4 + T cells (ELISPOT) (**e**), total CD4 + T cells (flow cytometry) (**f**) and late apoptotic CD4 + T cells (**g**) in the pLN of mock, CHIKV and CHIKV + PbA (*n* ≥ 5 per group) at 2, 4, 5 and 6 dpi. **e** Representative ELISPOT images showing spots generated by virus-stimulated CD4 + T cells isolated from 100,000 pLN cells (4 and 5 dpi) and 200,000 pLN cells (6 dpi) in CHIKV-infected and co-infected mice. **g** Representative dot plots displaying late apoptotic CD4 + T cells gating on 6 dpi. Late apoptotic CD4 + T cells are defined as CD45 + CD3 + CD4 + FVD + AnnexinV + cells. Data shown are pooled from four independent experiments. Data (**e**–**g**) from the co-infected group were compared against CHIKV-infected controls at the respective time-point using Mann-Whitney two-tailed test (*P *<* 0.05 and **P *<* 0.01). Data represent the means ± SD. Abbreviations: CHIKV, Chikungunya virus; dpi, days post infection; PbA, *Plasmodium berghei*-ANKA

To first determine whether co-infection could impair CD4^+^ T-cell expansion via the lymph nodes and disrupt infiltration into the joints, LTα^-/-^ mice that are devoid of lymph nodes^32^ were assessed. Within the context of concurrent co-infection, the spleen was not of primary focus as co-infection in splenectomised mice still reduced peak joint swelling when compared to CHIKV infected splenectomised mice (Fig. 4g). First, absence of lymph nodes delayed CHIKV-induced peak joint swelling from 6 dpi to 7–8 dpi (Fig. 6a). LTα^-/-^ mice with concurrent CHIKV + PbA co-infection showed equivalent joint swelling as CHIKV-infected only LTα^-/-^animals with no effect on viraemia (Fig. 6a, b). Lack of lymph nodes also increased tissue viral load and caused extensive CHIKV dissemination from 6 dpi onwards in CHIKV-infected mice (Fig. 6c, d and Supplementary Movie 3). Concurrent co-infected LTα^-/-^ mice still displayed an increased viral load and viral dissemination in the tissues from 10–18 dpi as compared to CHIKV-infected only LTα^-/-^animals (Fig. 6c, d and Supplementary Movie 3).

### Co-infection induces CD4 + T cells apoptosis in the pLN

The popliteal lymph node (pLN) is the nearest lymph node to the site of CHIKV infection in the footpad. As immune regulation leading to suppressed joint swelling is dependent on the lymph node (Fig. 6a), expansion kinetics of CD4^+^ T cells in the pLN during concurrent co-infection was profiled. At 4 dpi, when pathogenic CD4^+^ T-cell expansion is observed, CHIKV-specific CD4 + T cells numbers were similar between the pLN of CHIKV-infected and *Plasmodium* + CHIKV co-infected mice (Fig. 6e). These data imply similar levels of CD4^+^ T-cell expansion in the early stages of infection (4 dpi).

Interestingly, co-infection reduced the numbers of total and CHIKV-specific CD4^+^ T cells in the pLN at 5 and 6 dpi (Fig. 6e, f). *Plasmodium* is known to induce T-cell apoptosis in the secondary lymphoid organs^33,34^. As early CD4^+^ T-cell expansion (4 dpi) was similar between single and co-infected mice, it is plausible that *Plasmodium-*induced apoptosis may reduce CD4^+^ T-cell numbers in the pLN of co-infected mice at 5 and 6 dpi. As expected, co-infection resulted in a higher proportion (~ 2–3 folds) of late apoptotic CD4^+^ T cells in the pLN at 5 and 6 dpi (Fig. 6g).

The reduction of CD4^+^ T cells from 5 dpi in the pLN on joint inflammation would depend on the timing of migration of pathogenic CD4 + T cells to the infected tissue. Therefore, CHIKV-specific CD4^+^ T-cell migration kinetics was assessed in the joints by flow cytometry and IFNγ ELISPOT assay. The majority of CHIKV specific CD4 + T cells infiltrated the joint between 5 and 6 dpi (Supplementary Fig. 9), thus occurring after *Plasmodium*-induced CD4^+^ T-cell apoptosis. Together, the reduction of peak joint swelling in co-infected mice is associated with the elevation apoptotic of pathogenic CD4^+^ T cells in the lymph nodes prior to their migration into the joints.

### Co-infection limits CXCR3-mediated infiltration into joints

**Fig. 7:**
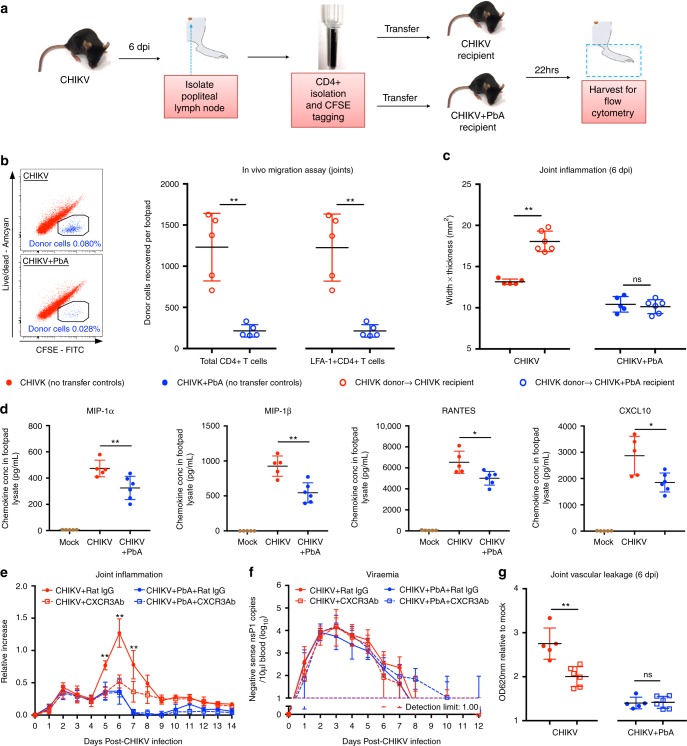
Co-infection abrogates CD4 + T-cell migration into the joints. **a** CD4 + T-cell in vivo migration assay. **b** In vivo migration assay showing number of recovered CHIKV-infected donor pLN CD4 + T cells (pooled) in the joints of CHIKV-infected or CHIKV + PbA-infected recipients (all groups *n* = 5). Representative dot plots displaying recovered donor-cell gating in 10,000 CD45 + cells in the footpad. **c** Joint inflammation of CHIKV (no transfer controls), CHIKV donor transferred to CHIKV recipient, CHIKV + PbA (no transfer controls) and CHIKV donor transferred to CHIKV + PbA recipient (all groups *n* ≥ 5). **d** Concentration of MIP-1a, MIP-1b, RANTES and CXCL10 in the joint-footpad cell lysate of mock (*n* = 5), CHIKV (*n* = 5) and CHIKV + PbA (*n* = 6) at 6 dpi. **e** Joint inflammation and **f** viraemia of CHIKV + Rat IgG (*n* = 6), CHIKV + CXCR3Ab (*n* = 6), CHIKV + PbA + Rat IgG (*n* = 6) and CHIKV + PbA + CXCR3Ab (*n* = 8). **g** Joint vascular leakage for CHIKV + Rat IgG, CHIKV + CXCR3Ab, CHIKV + PbA + Rat IgG and CHIKV + PbA + CXCR3Ab (all groups *n* ≥ 6). Joint vascular leakage was determined by Evan’s blue assay. All data were analyzed by Mann–Whitney two-tailed test (*ns*; not significant, *P *<* 0.05 and **P *<* 0.01). Each data point in the dot plot corresponds one mouse. Data represent the means ± SD. Abbbreviations: CHIKV, Chikungunya virus; dpi, days post infection; ns, not significant; PbA, *Plasmodium berghei*-ANKA

To assess whether reduced CD4^+^ T-cell numbers in the joints of co-infected mice could also be mediated by altered migration kinetics from the pLN in addition to increased levels of apoptosis, an in vivo migration assay was performed (Fig. 7a). This assay allows for the detection of any differences in cellular migration independent of the effects of increased *Plasmodium*-induced apoptosis at 5 dpi.

Concurrent *Plasmodium* and CHIKV co-infection inhibited migration of donor CD4^+^ T cells towards the joints of co-infected recipients by ~6-fold (Fig. 7b). Furthermore, the increase of joint swelling induced by donor’s CD4^+^ T cells in single CHIKV-infected recipients were absence in co-infected recipients (Fig. 7c). Immune mediators quantified from joint-footpad lysates at 6 dpi demonstrated a reduction of CCR5 ligands (MIP-1α, MIP-1β, RANTES) and CXCR3 ligand (CXCL10) in the joints (Fig. 7d).

CHIKV infection in CCR5^-/-^ mice and in WT mice with CXCR3 blockade was then performed to define the role of these two T-cell chemokine receptors. Joint swelling was suppressed at 2 dpi in CHIKV-infected CCR5^-/-^ mice, but no effect was observed during peak swelling (mediated by CD4^+^ T cells) from 5–8 dpi (Supplementary Fig. 10a). No effect on viraemia was observed in CHIKV-infected CCR5^-/-^ mice (Supplementary Fig. 10b). Conversely, CXCR3 blockade suppressed CHIKV-induced peak joint swelling from 5 to 7 dpi and reduced joint vascular leakage at 6 dpi with no effect on viraemia (Fig. 7g and Supplementary Fig. 10c, d). Importantly, CXCR3 blockade was associated with the inhibition of CD4^+^ T-cell infiltration into the joints (Supplementary Fig. 10e). However, CXCR3 blockade in concurrent *Plasmodium* and CHIKV co-infected mice did not significantly reduce joint swelling or vascular leakage at 6 dpi as compared to co-infected mice with no blockade (Fig. 7e, g). CXCR3 blockade has minimal effect on viraemia (Fig. 7f). Together, these results show that CXCR3 and not CCR5, is functionally important for CD4^+^ T-cell migration during CHIKV infection, and co-infection sufficiently abrogates CXCR3-mediated joint swelling.

## Discussion

In this study, a CHIKV mouse model that recapitulates the parameters of CHIKV infection in human patients (in terms of viral load profile and joint pathology such as tenosynovitis, myositis, oedema, and cellular infiltration^21,22,24,35,36^) was used to understand the effects of *Plasmodium* co-infection on virus-induced pathology. We showed that *Plasmodium* infection modulates early IFNγ production, B-cell maturation and CD4 + T cell responses toward CHIKV to suppress acute joint pathology and delay tissue viral resolution in the co-infected host.

We found that elevated IFNγ production, induced by prior acute *Plasmodium* infection, exerts an anti-viral effect, abrogating systemic viral load and suppressing early virus replication in the joint tissue, leading to abolished joint disease. *Plasmodium-*induced IFNγ is produced by innate cellular subsets, including NK, NKT cells and γδ T cells^37^. We postulate that acute *Plasmodium* infection triggers high IFNγ production^23^ that could “prime” cells to suppress initial viral replication in CHIKV-target cells such as fibroblasts. This effect would lead to the observed early reduction in viral load locally in the joints. Conversely, viral suppression in the monocytes/macrophages could also limit early viral dissemination to other tissues.

Disruption of germinal center architecture and germinal center B-cell maturation has been documented in experimental mouse and non-human primate models of *Plasmodium*^25–29^. Disturbance of B-cell homeostasis has been reported in *Plasmodium*-infected patients^38^. Consistent with previous studies^25–29^, *Plasmodium* co-infection delays early (6 dpi) generation of GC B cells (B220^+^GL7^+^CD95^+^CD38^−^), which leads to reduced GC-dependent CD73^+^ memory B cells at a later stage of infection (15 dpi)^31^. This disruption to GC B-cell formation was originally proposed to be mediated by impaired T follicular helper-cell differentiation during *Plasmodium* infection^26^. Here, we found that changes to the splenic B-cell phenotype is associated with inefficient CHIKV-neutralizing antibody generation, delayed virus resolution in the joints and elevated virus dissemination during chronic stages of CHIKV infection. These changes in viral load in the joints of co-infected mice did not aggravate joint pathology at the earliest time-point of viral resolution in co-infected mice (45 dpi). Nonetheless, such disruption to the B-cell humoral response during co-infection may negatively impact the recall response against secondary CHIKV infection.

Pathogenic virus-specific CD4^+^ T cells are the primary mediator of the peak of CHIKV-induced joint pathology^20,35^. Induction of Th1 cytokines in both CHIKV-infected patients and mice suggests that CD4^+^ T cells mediate joint pathology through a Th1-mediated mechanism^36,39–43^. In fact, co-infection suppresses peak joint pathology of CHIKV infection by limiting the infiltration of pathogenic virus-specific IFNγ (Th1)-producing CD4^+^ T cells in the joints. Here, suppression is mediated in two stages. *Plasmodium-* infection induces apoptosis of a fraction of CD4^+^ T cells in the lymph node early after priming, and abrogates migration of the remaining live CD4^+^ T cells by suppressing chemotactic factors in the joints.

Prior to this study, the chemokines that regulate CD4^+^ T-cell migration during CHIKV infection were unknown. We show here that CXCR3, but not CCR5, is a critical mediator of CD4 + T-cell migration into infected joints. CXCR3 blockade in co-infected mice did not reduce joint swelling further, suggesting that the CXCR3-mediated pathway is already suppressed in co-infected mice. Suppresion of joint swelling was more pronounced in co-infected mice with or without CXCR3 blockade as compared to CHIKV-infected mice with CXCR3 blockade. This finding suggests that suppression of CHIKV-induced joint swelling in co-infected mice is only partially dependent on CXCR3.

These findings of chikungunya protection during co-infection with *Plasmodium* have important implications when considering malaria intervention programmes. Effective malaria control programmes have declined the global incidence of malaria over the past decade^10,44^ and this is associated with the increasing trend of chikungunya incidence over a similar period of time^8^. Interestingly, abrogation of systemic viral load during co-infection would lower vector competence, as lower blood viral titres would reduce the proportion of vectors being successfully infected^45^. On the other hand, in the context of uncontrolled *Plasmodium* infection, the reduction of severe joint pathology by malaria co-infection would likely lower or mask CHIKV-related healthcare burden and prevalence. As the World Health Organisation scales up malaria intervention programmes to aggressively decline the incidence of malaria^46^, the severity and numbers of CHIKV clinical cases, and vector competence, could indirectly increase. Therefore, alphavirus management should be integrated as part of the ongoing malaria intervention programme.

## Methods

### Mice

Male and female WT, *CCR5*^*−/−*^*, LTα*^−*/*−^, and *IFNγ*^−*/*−^ mice (aged 5–7 weeks) were maintained on a C57BL/6J background. All experiments were age and gender matched. All mice were bred and housed under specific pathogen-free conditions in the Biological Resource Centre, Agency for Science, Technology and Research, Singapore. All experiments and procedures were approved by the Institutional Animal Care and Use Committee (IACUC: 140968) of the Agency for Science, Technology and Research, Singapore, in accordance with the guidelines of the Agri-Food and Veterinary Authority and the National Advisory Committee for Laboratory Animal Research of Singapore.

### Pathogens and infection

The lethal *P. berghei* ANKA (PbA) clone 15Cy1 was used to induce experimental cerebral malaria (ECM)^47^. The *P. yoelii yoelii* 17XNL clone 1.1 (Py17x) was used to induce non-lethal malaria^48^. Infected red blood cells (iRBC) were prepared by in vivo serial passage in C57BL/6J mice and blood samples stored in Alsever’s solution in liquid nitrogen.

The CHIKV strain (SGP11) was previously isolated from blood samples taken from patients with CHIKV infection, admitted to the National University Hospital Singapore during the 2008 outbreak in Singapore^49^. The CHIKV variant expressing firefly luciferase (CHIKV-luc) or Zs-Green was constructed using a full-length infectious cDNA clone of the viral isolate CHIKV strain LR2006 OPY1^50^. CHIKV was propagated in *Aedes albopictus* C6/36 cultures (ATCC^®^CRL-1660^TM^) and quantified by standard plaque assay in Vero E6 cells (ATCC^®^ CCL-81^TM^) as plaque forming units (PFU)^20^.

Mice were infected with PbA by intraperitoneal (i.p.) injection of 10^6^ iRBC and with CHIKV by subcutaneous inoculation of 10^6^ PFU CHIKV (in 30 μl PBS) in the ventral side of the right hind footpad towards the ankle.

### CHIKV disease monitoring in mice

Viraemia was monitored daily from 1 day post-infection (dpi) until 8 dpi, and subsequently on every alternate day until 14 dpi. Briefly, 10 μL of blood collected from the tail vein was diluted in 120 μL PBS and 10 μL citrate-phosphate-dextrose solution (Sigma-Aldrich). Viral RNA was extracted with QIAmp Viral RNA kit (Qiagen) in accordance to manufacturer’s protocol. To measure the levels of replicating virus the extracted samples, RNA copies of negative sense *nsP1* were quantified using quantitative RT-PCR. Primers and probe sequences were adapted from Plaskon et al.^51^ to target the same region of the negative sense *nsP1* of the strain of CHIKV used in this study. The sequences of *nsP1* forward and reverse primers are 5′GGCAGTATCGTGAATTCGATGCGACACGGAGACGCCAACATT3′ and 5′AATAAATCATAAGTCTGCTCTCTGTCTACATGA3′. The sequence of probe used is 5′TGCTTACACACAGACGT3′. Both primers and probe targeting this region of the negative sense *nsP1* has been previously validated to be highly strand specific^51^. To note, both free viruses and viruses present inside peripheral leukocytes were determined. Comparison of viral load using paired whole blood and serum samples shows that a significant portion of viral RNA would have been missed if measurement was only done using plasma or serum (Supplementary Fig. 11).

Joint swelling of the footpad was scored daily from 0 to 14 dpi^20^. Measurements were done for both height (thickness) and breadth of the foot and were quantified as [height x breadth]. Degree of inflammation was expressed as relative increase in footpad size as compared to pre-infection (day 0) with the following formula: [(x−day 0)/day 0], where x is the quantified footpad measurement for each respective day.

Viral load and dissemination of luciferase-tagged virus (CHIKV-luc) into the organ tissues was tracked using an in vivo bioluminescence imaging system (IVIS; Perkin Elmer)^20^. Luciferase substrate, D-luciferin potassium salt (Caliper Life sciences), was dissolved in PBS at a concentration of 5 mg/ml. Mice were shaved and anesthetized in an oxygen-rich induction chamber with 2% isoflurane. Measurements were performed 2 min after subcutaneous injection of 100 μl of luciferin solution. Whole body imaging was performed with the animal in a ventral position. Foot imaging was performed with the animal in a dorsal position. Bioluminescence imaging was acquired with a field of view (FOV) of 21.7 cm and 13.1 cm for whole body (FOV-D) and foot (FOV-C) respectively. Exposure condition was an initial 60 s, followed by a 4 min delay before another exposure at 60 s. In the event that luminescence readings were above the detection limit of machine, the exposure time was reduced and kept consistent across groups. Bioluminescence signals taken pre-infection (0 dpi) were used for background subtraction. For bioluminescence quantifications, regions of interest were drawn using the software Living Image 3.0, and average radiance (p/s/cm^2^/sr) was determined.

### Viral load determination in footpad

Mice were anesthetised with ketamine-xylazine (150 mg/kg of ketamine; 10 mg/kg of xylazine), perfused intracardially with PBS and organs were harvested and preserved in 1 mL TRIZOL (Invitrogen) at –80 °C. Tissue samples were transferred to gentleMACS M Tubes (Miltenyi Biotec) and homogenized using a rotor-stator homogenizer (Xiril Dispomix)^52^. Homogenized tissues were transferred to clean Eppendorf tubes and mixed thoroughly with 230 μL of chloroform, and allowed to stand for 2 min in room temperature. Tissue mixtures were centrifuged at 12 000 g for 10 min at 4 °C. The aqueous phase was collected into a clean Eppendorf tube, and an equal volume of 70% ethanol was added in and mixed. Total RNA was subsequently extracted with RNeasy kit (Qiagen) following manufacturer’s protocol. RNA copies of negative sense *nsP1* were subsequently quantified using quantitative RT-PCR.

### *Plasmodium* disease monitoring in mice

Parasitaemia was monitored by flow cytometry daily from 3 dpi to 12 dpi and subsequently on alternate days^53^. Briefly, 1 μl of blood was collected in 100 μl of PBS and stained in 1 μL of Hoechst (800 µM; Sigma), 0.5 μl of dihydroethidium (1 μg/ml; Sigma), 0.5 μl of PBS and 2 μl of monoclonal antibody CD45 coupled to Allophycocyanine (Miltenyi Biotech; Cat no. 130-102-544). The blood preparation was incubated for 20 min before addition of 400 μl PBS. Stained samples were acquired on a LSR II flow cytometer (BD Biosciences) using BD FACSDiva software and analyzed using Flowjo software. *Plasmodium* infected RBCs were determined as Hoechst + /dihydroethidium + /CD45- cells.

### In vivo IFNγ neutralization and CXCR3 blockade

Anti-mouse IFNγ (0.5 mg; clone XMG1.2; Bioxcell; Cat no. BE0055) was administered on −4, −2, 0 and 2 dpi via i.p injection. Anti-mouse CXCR3 (0.5 mg; clone CXCR3-173; Bioxcell; Cat no. BE0249) was administered on 0, 2, 4 and 6 dpi via i.p. injection. Control groups were given Rat IgG (Sigma Aldrich) at similar dosage and timing as treatment groups.

### Splenectomy

Mice aged 4-weeks old were anesthetised with 80 μl ketamine:xylaxine (150 mg/kg:10 mg/kg). Incisions were made on the left flank, and the primary artery connecting the spleen to the other organs located within the fat was sealed. Post-surgery, enrofloxacin (5 mg/kg) and PBS (1 ml) was given and mice were monitored for recovery. A control, sham operation included similar incisions to the skin, without spleen removal and similar antibiotic treatment post-surgery. ~ 5 weeks were given for mice to completely recover from surgery prior to further experiments, hence infection in these mice were delayed to 9 weeks old.

### Histology of joint footpad tissues

Mice were euthanised by perfusion with PBS post anesthesia and tissues were harvested and fixed in 10% neutral buffered formalin, and cut into 5 μm-thick sections for hematoxylin and eosin (H&E) staining^35^. Histopathological examination was carried out in a blinded fashion based on the presence of oedema, inflammation, muscle necrosis, tendonitis, and synovitis in mouse joint. Severity of grading was assigned on the following scale: 1—minimal; 2—mild; 3—moderate, 4—marked & 5—severe as previously described^21^.

### Immunohistochemistry (IHC) and image analysis

Immunohistochemical staining of the joint-footpad was performed using a polyclonal α-mouse CD3 antibody (1:200 dilution; Dako; Cat no. A0452), as previously described^35^. Stained sections were scanned using a Leica SCN400 slide scanner (Leica Microsystem GmbH, Germany) at a 20x magnification and exported to the SlidePath Digital Image system (Leica microsystem GmbH, Germany). CD3 expression was analyzed using cytoplasmic/membranous algorithms within the Leica SlidePath Tissue Image Analysis (tissue IA) software. The results among the different groups were computed and analyzed for statistical significance.

### Quantification of joint vascular leakage by tracer assay

A Tracer 653 Assay (Molecular Targeting Technology, Inc) was performed as previously described^36^. Briefly, 100 μl tracer solution was injected (i.v) into each mouse, and the tracer solution that entered the joints was quantified using the IVIS. Regions of interest were drawn using Living Image 3.0 software, and average radiance [Log_10_(p/s/cm^2^/sr)/(μW/cm^2^)] was determined.

### Leukocyte profiling in the joints, pLN and spleen

Joints, pLN and spleen were surgically extracted and processed to obtain a homogenous cell suspension. For spleens, tissue was dissociated in RPMI medium containing 10% FBS (complete RPMI) and passed through a 40 μm cell strainer (Fisherbrand), followed by RBC lysis with RBC lysis buffer (R&D system). For pLN, extracted tissue was incubated in 1 mL of digestion medium [dispase (2 U/mL; Invitrogen), collagenase IV (20 mg/mL; Sigma-Aldrich), and DNase I mix (50 mg/mL; Roche Applied Science) in complete RPMI medium] for 30 min at 37 °C. Cells were passed through 70 μm nylon mesh cloth (Sefar) followed by RBC lysis with RBC lysis buffer. For footpad, tissue were removed,de-skinned in 4 ml of digestion medium and incubated for 3 h at 37 °C. Digested tissues were passed through a 40 μm cell strainer, followed by RBC lysis. Cells were then resuspended in 1 ml of complete RPMI and overlay on 35% v/v Percoll (Sigma-Aldrich)/RPMI medium, centrifuged at 1024 g for 20 min.

Isolated cells were stained with LIVE/DEAD Aqua (Life Technologies), then blocked in 100 µl blocking buffer consisting of a mix of 1% rat and mouse serum (Sigma-Aldrich) in FACS buffer [1% BSA, 2 μm EDTA in PBS]. Next, cells were stained with conjugated antibodies for 20 min and fixed in IC fixation buffer (ebioscience) for 5 min before acquisition using a LSR II flow cytometer (BD Biosciences). Conjugated antibodies used were: α-CD45 (1:400 dilution; clone 30-F11, BD Biosciences; Cat no. 557659), α-CD3 (1:200 dilution; clone 17A2, BD Biosciences; Cat no. 560591), α-CD4 (1:400 dilution clone GK1.5, Biolegend; Cat no. 12-0041-83), α-CD8 (1:400 dilution; clone 53–6.7, BD Biosciences; Cat no. 100743), α-LFA-1 (1: 200 dilution; clone H155-78; Biolegend; Cat no. 141008), α-NK1.1 (1:400 dilution; clone PK136, ebioscience; Cat no. 17-5941-82), α-CD11b (1:400 dilution clone M1/70, Biolegend; Cat no. 101239), α-CD11c (1:400 dilution; clone N418, Biolegend; Cat no. 117333), α-Ly6C (1:400 dilution; clone AL-21, BD Biosciences; Cat no. 560596), α-Ly6G (1:400 dilution; clone 1A8, Biolegend; Cat no. 127612), α-B220 (1:400 dilution; clone RA3-6B2; Biolegend; Cat no. 103230), α-GL7 (1:400 dilution; clone GL7; Biolegend; Cat no. 144607), α-CD95 (1:200 dilution; clone 3F1; BD Biosciences; Cat no. 743255), α-CD38 (1:200 dilution; clone 90; Biolegend; Cat no. 102717), α-CD73 (1:200 dilution; clone TY/11.8; Thermofisher Scientific; Cat no. 46-0731-82), α-CD138 (1:200 dilution; clone 281-2; BD Biosciences; Cat no. 563147), α-IgD (1:200 dilution; clone 11–26 c.2a; BD Biosciences; Cat no. 565348) and α-CD80 (1:400 dilution; clone 16-10A1, Biolegend; Cat no. 104703). Representative gating strategy is shown in Supplementary Fig. 12.

### Quantification of apoptosis

Apoptosis of isolated pLN cells was determined by Annexin V assay (eBioscience; Cat no. 88-8005-72) according to the manufacturer’s protocol. Briefly, 10^6^ pLN cells underwent surface staining of for 20 min followed by staining with fixable viability dye (1:500 dilution; FVD; eBioscience; Cat no. 65-0866-14) for 30 min on ice. Cells were then stained with FITC-conjugated Annexin V for 15 min followed by acquisition using a LSR II flow cytometer. Late apoptotic cells were defined by FVD + /Annexin V + staining. Representative gating strategy is shown in Supplementary Fig. 13.

### Quantification of CHIKV-specific CD4 + T cells by ELISPOT

IFNγ ELISPOT assays (MABTECH; Cat no. 3321-2 A) were conducted using isolated CD4^+^ T cells from 100,000 pLN or footpad cells were stimulated with 5 × 10^6^ virions and 200,000 naïve splenocytes following manufacturer’s protocol. Cells were stimulated with the virus in RPMI medium supplemented with 10% FBS and 30 U/mL IL2 (Sigma-Aldrich) for 18 h. The number of spots was quantified using an ImmunoSpot 5.0 Analyzer Professional DC (Cellular Technology Ltd), and the data were back calculated as the number of virus-specific CD4 + T cells per organ.

### Quantification of CHIKV-specific IgM and Total IgG

A virion-based ELISA was used to quantify IgM and total IgG levels in the sera^24^. CHIKV-coated (10^6^ virions/ well in 50 mL PBS) polystyrene 96-well MaxiSorp plates (Nunc) were blocked with PBS containing 0.05% Tween 20 (PBST) and 5% w/v nonfat milk for 1.5 h at 37 °C. Sera from test groups were heat-inactivated and diluted in antibody diluent (0.05% PBST + 2.5% w/v nonfat milk) at 1:200 and 1:2000 for IgM and total IgG quantification respectively. 100 μl of diluted sera per well was added and allowed to incubate for 1 h at 37 °C. Subsequently, test sera were washed off and 100 μl of secondary antibody, horseradish peroxidase-conjugated goat anti-mouse IgG (1:20,000 dilution; Santa Cruz; Cat no. SC-2031) or IgM (1:10,000 dilution; Santa Cruz; Cat no. SC-2064) were added and allowed to incubate for 30 min. ELISA assays were developed using Tetramethylbenzidine (TMB) substrate and terminated with Stop reagent (Sigma-Aldrich). Absorbance was measured at 450 nm. IgM and Total IgG were quantified at 1:200 and 1:2000 dilutions, respectively.

### Sero-neutralization assay

The neutralizing activity of sera was tested in a modified fluorescence-based cell-infection assay in HEK293T (ATCC^®^CRL-3216™) cells (2 × 10^4^ cells/well) of a 96-well plate^24^. Briefly, Zs-Green tagged CHIKV was incubated with various dilutions of heat-inactivated mouse sera at a multiplicity of infection (MOI) of 8 for 2 h at 37 °C with gentle agitation. Virus–antibody (Ab) mixtures were then added to HEK293T cells and incubated for a further 1.5 h at 37 °C. Virus–Ab overlays were subsequently removed and the cells were re-incubated in fresh DMEM supplemented with 10% FBS for 15 h at 37 °C before fixation with 4% paraformaldehyde, DAPI (0.5 μg/mL) staining and quantification using the Cellomics ArrayScan V (Thermo Fisher Scientific, Waltham, MA) or MACSQuant analyser 10 (Miltenyi Biotec). The percentage of infectivity was calculated as follows: % Infectivity = 100 × (%responder from sero-neutralization group/% responder from virus infection group).

### In vivo migration assay

At 6 dpi, pLN from singly CHIKV-infected donors were harvested. Total CD4 + T cells were isolated from these donors through negative selection using a CD4 + T-cell isolation kit (Miltenyi Biotec). Cells were subsequently labeled with 5 μM CFSE solution (Sigma-Aldrich) for 10 min, washed with PBS. A solution of 2 × 10^6^ cells in 200 μl PBS was injected (i.v.) into a CHIKV only or co-infected recipient at 5 dpi. A portion of these donor cells were stained with α-CD45, α-CD3, α-CD4, α-CD8, and α-LFA-1 antibodies according to dilutions specified above and analyzed by flow cytometry to profile the numbers of total and LFA-1 + CD4 + T cells in 2 × 10^6^ donor cells. The footpads were extracted from recipient mice 22 h after transfer, and processed to obtain a homogenous cell suspension. Extracted cells were stained with Live/Dead determination dye (Invitrogen) for 20 min, followed by α-CD45, α-CD3, α-CD4, α-CD8, and α-LFA-1 antibodies. The donor cells were identified by FITC expression using flow cytometry. The migratory capacity of the donor cells towards the joint-footpad was determined by the ratio of donor cells obtained from the recipient footpad divided by initial number of donor cells transferred.

### Cytokines/chemokines quantification

Footpads were extracted and homogenized in RIPA buffer (50 mM Tris-HCl pH 7.4; 1% NP-40; 0.25% Sodium deoxycholate; 150 mM NaCl; 1 mM EDTA) with 1x protease inhibitors (Roche) in a gentleMACS M tube using a gentleMACS Dissociator (Miltenyi). Cell lysates were then sonicated at 70% intensity for 15 s (Branson Ultrasonics Sonifier^TM^ S-450), and the supernatants were collected for cytokine/chemokine quantification using a 36 plex mouse ProcartaPlex^TM^ luminex assay (Cat no. EPX360-26092-901), according to manufacturer’s protocol. Data are expressed as pg/mL in the footpad lysate.

### Statistical analysis

Statistical analyses were performed according to the parametric or nonparametric distribution of the data using Prism 6 (GraphPad Software). Normality tests were performed by D’Agostino-Pearson omnibus normality test. All data with a normal distribution were analyzed by unpaired T-test or one-way ANOVA with Tukey’s *post-hoc* test. All data that did not meet the normality requirements were analyzed by Mann-Whitney 2-tailed analysis or Kruskal-Wallis with Dunn’s multiple comparisons. P values < 0.05 were considered statistically significant.

## Electronic supplementary material

Supplementary Information

Description of Additional Supplementary Files

Supplementary Movie 1

Supplementary Movie 2

Supplementary Movie 3

## Data Availability

The data that support the findings of this study are available from the corresponding author upon request.
