# Supplementary material for: Plasmodium co-infection protects against chikungunya virus-induced pathologies
Source: Nat Commun. 2018 Sep 25;9:3905. doi: 10.1038/s41467-018-06227-9 (PMC6156405; doi:10.1038/s41467-018-06227-9)
Supplement: Supplementary file 1 — Supplementary Information [file 41467_2018_6227_MOESM1_ESM.pdf]

Plasmodium co-infection protects against chikungunya virus-induced pathologies

Teo et al.

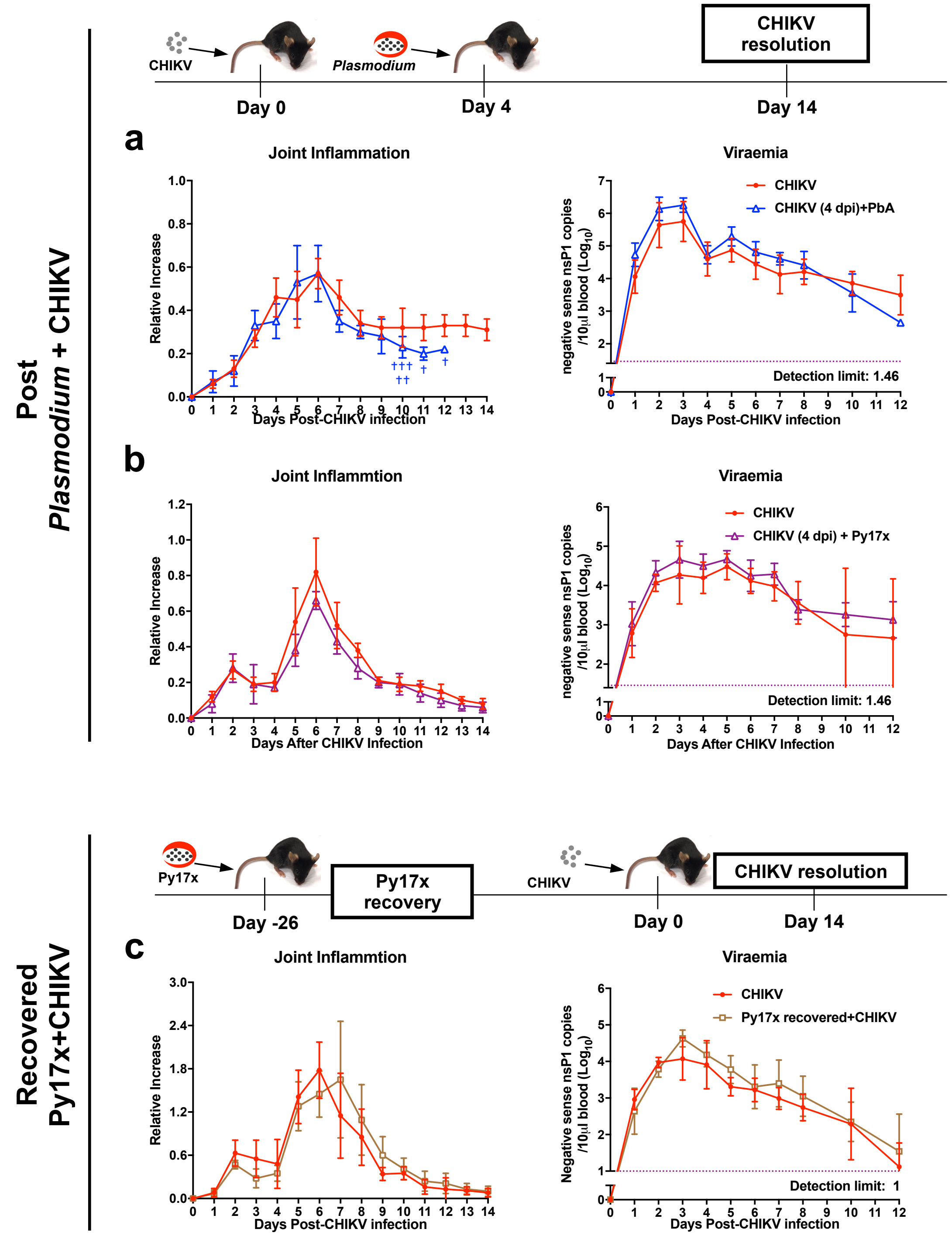

Supplementary Figure 1 I *Plasmodium* infection post CHIKV infection has no effect on CHIKV pathology. **a**, Joint inflammation and viraemia of CHIKV (n = 7) and CHIKV (4 dpi)+PbA (n = 7) groups. **b**, Joint inflammation and viraemia of CHIKV (n = 5) and CHIKV (4 dpi)+Py17x (n = 4) groups. For **a** and **b**, *Plasmodium* infection occurred 4 days post CHIKV infection, as shown in the schematic. Each blue "†" symbol represents one mouse that died of experimental cerebral malaria on the respective day as shown in **a** – joint inflammation. **c**, Joint inflammation and viraemia of CHIKV (n = 5) and Py17x recovered+CHIKV (n = 5) groups. CHIKV infection occurred after parasitaemia resolved at 26 days post Py17x infection. Data represent the means ± SD. Abbreviations: CHIKV, Chikungunya virus; dpi, days post infection; PbA, *Plasmodium berghei*-ANKA; Py17x, *Plasmodium yoelii* 17X.

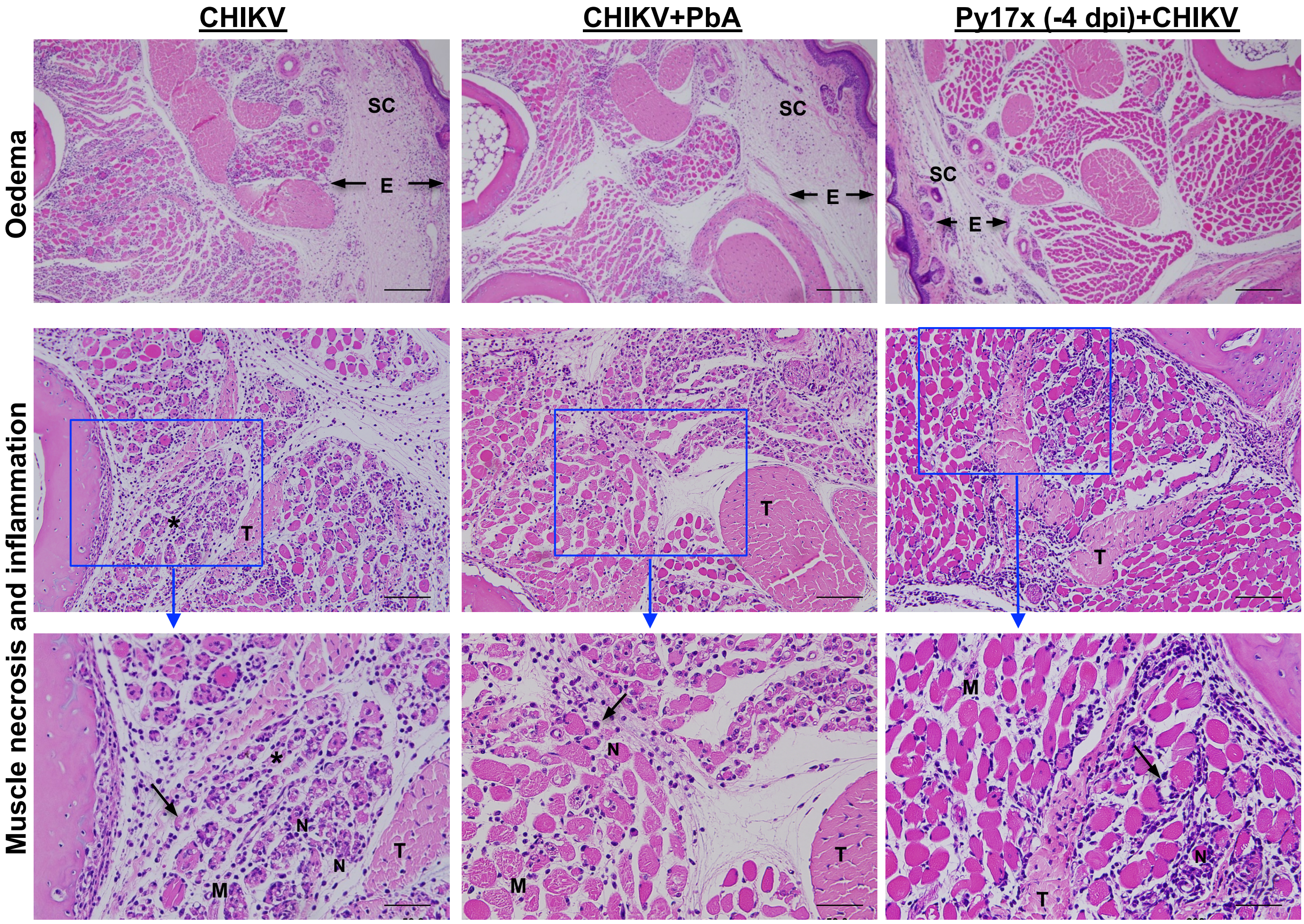

Supplementary Figure 2 I *Plasmodium* and CHIKV co-infection suppresses CHIKV-induced oedema, cellular infiltration and muscle necrosis in the joints. Representative haematoxylin and eosin (H&E) images of the inflamed joint footpad of CHIKV (*n* =6), CHIKV+PbA (*n* = 4) and Py17x (-4 dpi)+CHIKV (*n* =5) on 6 dpi used for histological assessment in figure 2h-j. E, region of edema; M, muscle; N, muscle necrosis; SC, subcutaneous region; T, tendon; *, region of extensive necrosis not present in co-infected groups; unlabeled black arrow, infiltration of mononuclear cells; blue box, region enlarged to higher magnification (below). Scale bar for Oedema is 200 μm. Scale bar for muscle necrosis and inflammation is 100 μm and 50 μm for blue insert. Abbreviations: CHIKV, Chikungunya virus; dpi, days post infection; PbA, *Plasmodium berghei*-ANKA; Py17x, *Plasmodium yoelii* 17X.

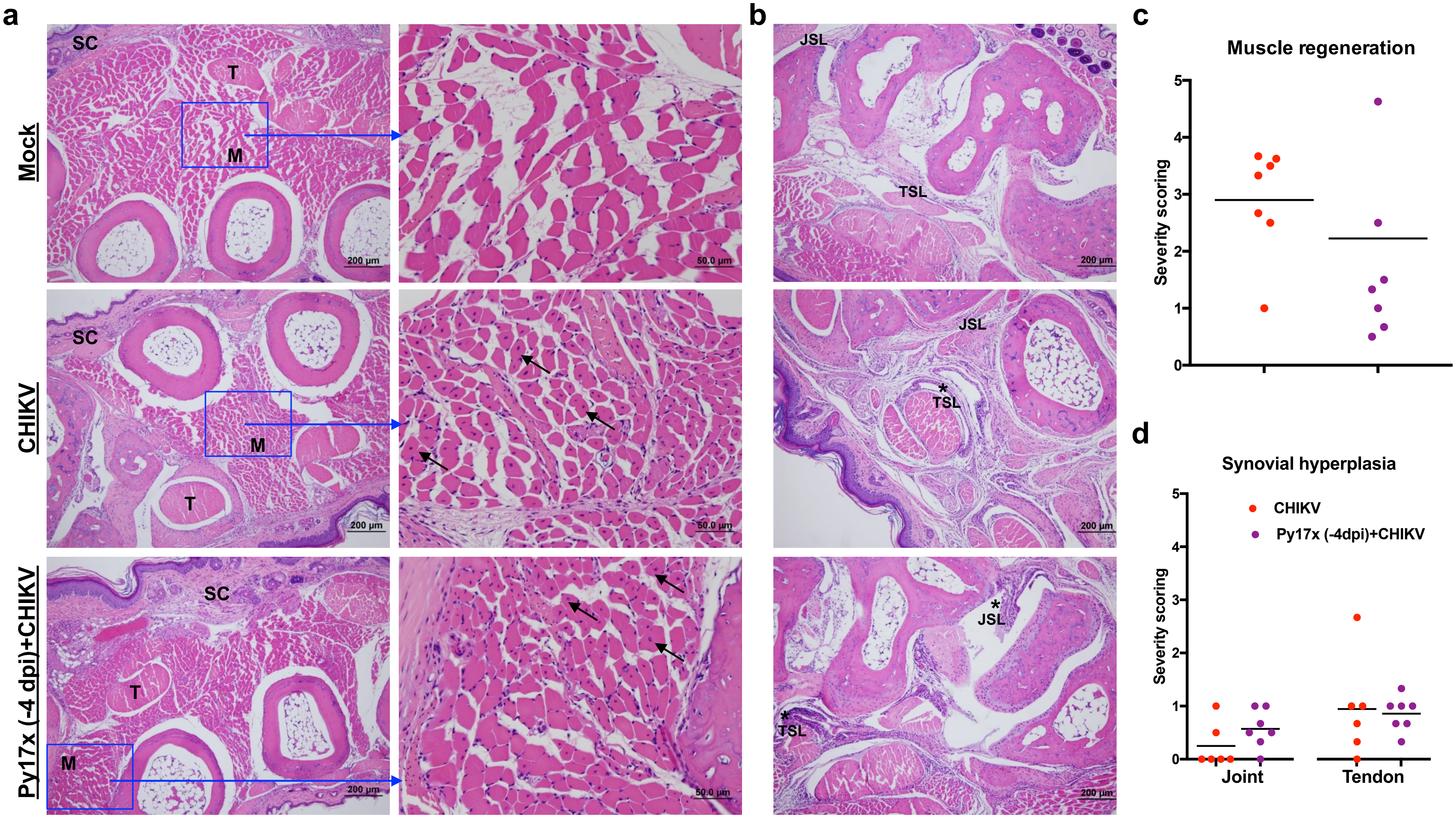

Supplementary Figure 3 I Joint phenotype of acute Py17x (-4 dpi) and CHIKV co-infection at 45 dpi. a, Representative haematoxylin and eosin (H&E) staining of joint sections displaying the subcutaneous region (SC), muscles (M) and tendon (T) in naïve, CHIKV and Py17x (-4 dpi)+CHIKV mice at 45 dpi. The blue box indicates the muscle regions taken at higher magnification, and examples of regenerative muscle identified by centrally placed nucleus are indicated by black arrows. Scale bar for main images are 200 μm and for blue inserts are 50 μm. **b**, Representative H&E images of the joint displaying the joint synovial lining (JSL) and tendon synovial lining (TSL) in naïve, CHIKV and Py17x (-4 dpi)+CHIKV mice at 45 dpi. Scale bar of images are 200 μm. Regions of hyperplasia in the JSL or TSL of infected mice that are absent in the naïve mice are indicated by *. Severity scoring of **c**, muscle regeneration and **d**, synovial hyperplasia in naive (n = 2), CHIKV (n = 6) and Py17x (-4 dpi) +CHIKV (n = 7) at 45 dpi. All scoring were relative to mock-infected mice, which were scored as zero. Abbreviations: CHIKV, Chikungunya virus; dpi, days post infection; Py17x, *Plasmodium yoelii* 17X.

Supplementary Figure 3_Teo *et al.*, 2018

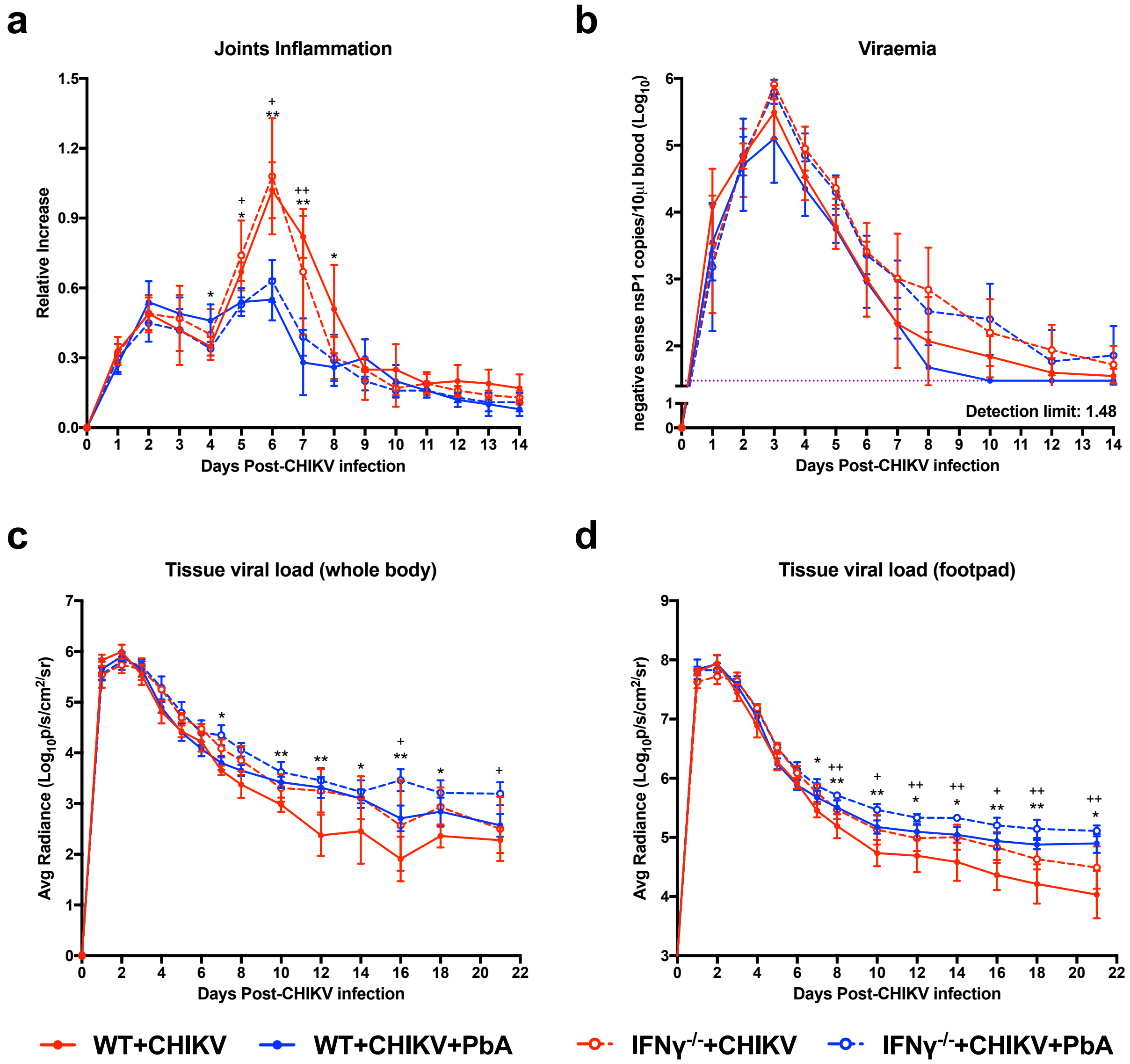

Supplementary Figure 4 I IFNγ is not a mediator for suppression of joint inflammation and delayed CHIKV resolution in tissues during concurrent CHIKV and *Plasmodium* co-infection. a, Joint inflammation, **b**, viraemia and viral load in the **c**, whole body and **d**, footpad of WT+CHIKV (n = 5), WT+CHIKV+PbA (n = 5), IFNγ+CHIKV (n = 5) and IFNγ+CHIKV+PbA (n = 5) groups. A Mann-Whitney two-tailed test was used to compare co-infected mice with their respective CHIKV-infected controls on the same genetic background. Comparisons between WT+CHIKV and WT+CHIKV+PbA are represented by $^{*}P < 0.05$ and $^{**}P < 0.01$; comparisons between IFNγ+CHIKV and IFNγ+CHIKV+PbA are represented by $^{+}P < 0.05$ and $^{++}P < 0.01$. Data represent the means ± SD. Abbreviations: CHIKV, Chikungunya virus; PbA, *Plasmodium berghei*-ANKA; WT, wild type..

Supplementary Figure 4_Teo *et al.*, 2018

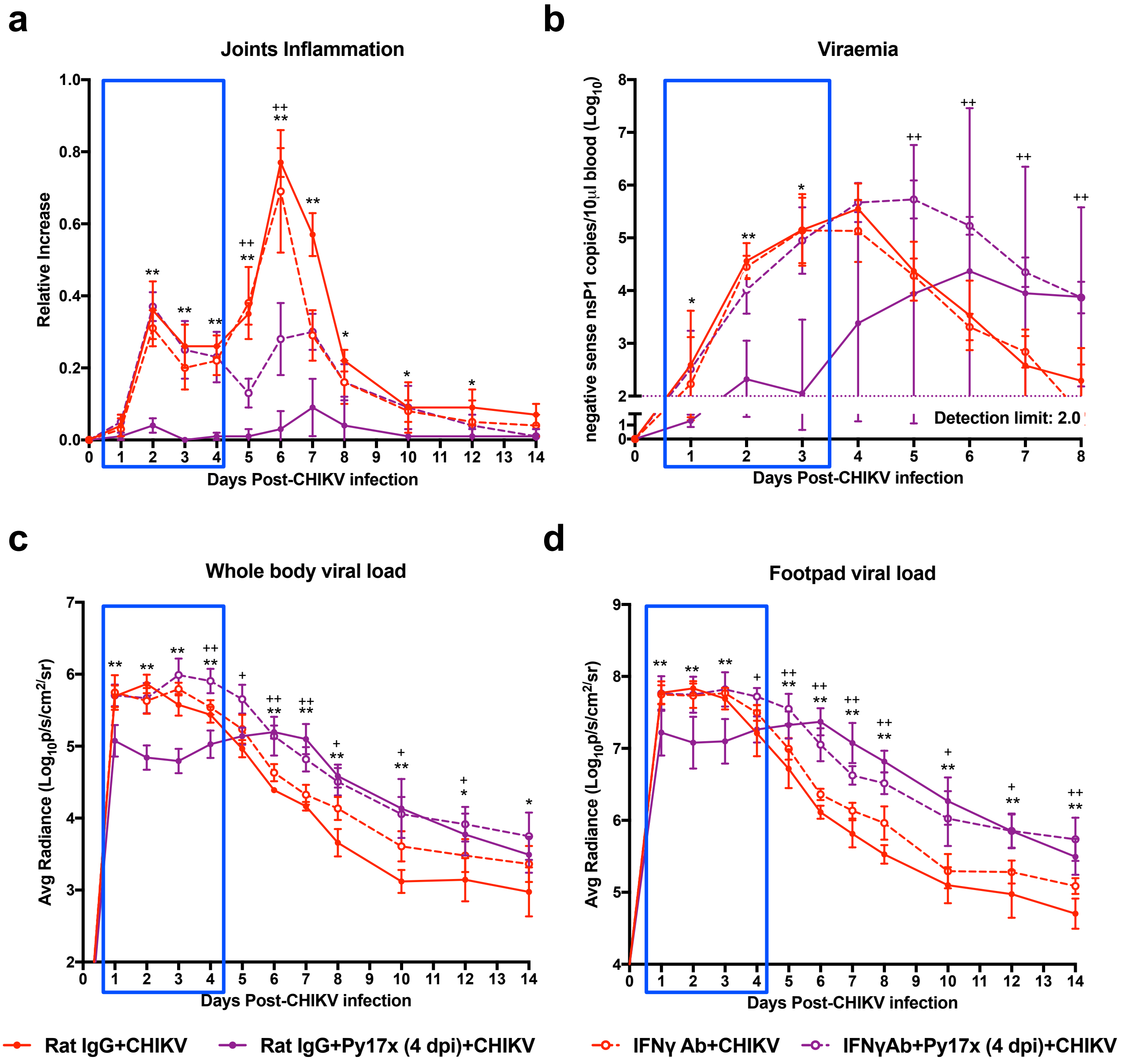

Supplementary Figure 5 I Disease kinetics with acute IFNγ neutralization during pre-Py17x (-4 dpi) and CHIKV co-infection. a, Joint inflammation, **b**, viraemia and viral load in the **c**, whole body and **d**, footpad of Rat IgG+CHIKV (*n* = 5), Rat IgG+Py17x (-4 dpi)+CHIKV (*n* = 5), IFNγ Ab+CHIKV (*n* = 5) and IFNγ Ab+ Py17x (-4 dpi) +CHIKV (*n* = 5) groups. Mice were treated with 0.5 mg of anti-IFNγ antibody or rat IgG on -4, -2, 0 and 2 dpi. Blue box denotes acute phenotypes (1 – 4 dpi) induced in Rat IgG+Py17x (-4 dpi)+CHIKV co-infected mice that are ameliorated with early IFNγ neutralisation. Mann-Whitney two-tailed test was used to compare CoIF mice against their respective CHIKV-infected controls that received the same antibody treatments. Comparisons between Rat IgG+CHIKV and Rat IgG+Py17x (-4 dpi)+CHIKV are represented by $^{*}P < 0.05$ and $^{**}P < 0.01$; comparisons between IFNγ Ab+CHIKV and and IFNγ Ab+ Py17x (-4 dpi)+CHIKV are represented by $^{+}P < 0.05$ and $^{++}P < 0.01$. Data represent the means ± SD and are representative of two independent experiments. Abbreviations: CHIKV, Chikungunya virus; dpi, days post infection; Py17x, *Plasmodium yoelii* 17X.

Supplementary Figure 5_Teo *et al.*, 2018

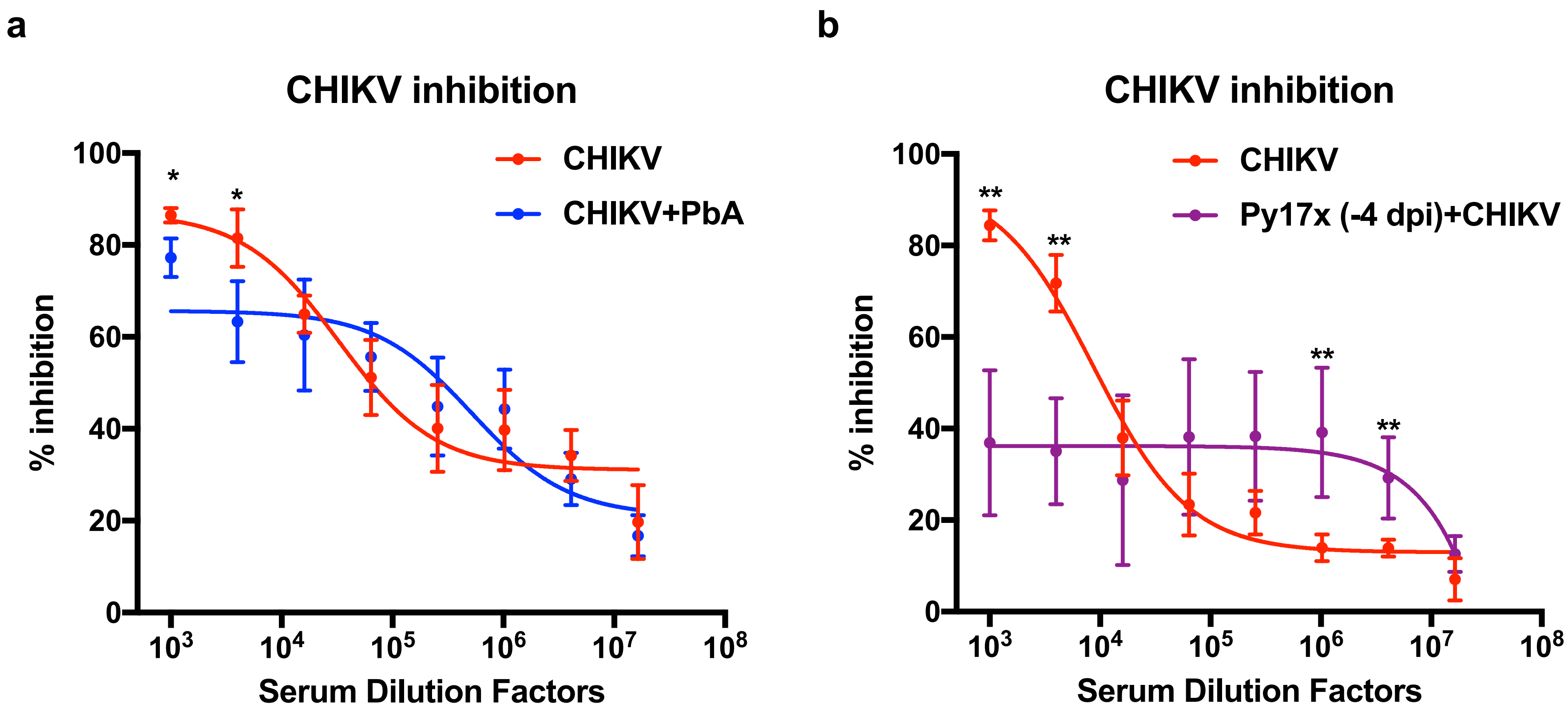

Supplementary Figure 6 I Inhibition of CHIKV infection by 15 dpi sera from PbA concurrent co-infection and Pre-Py17x (-4 dpi) co-infection. Neutralization assays were performed using 15 dpi sera from infected animals. %inhibition was calculated by 100% - relative infectivity. **a**, Inhibition of CHIKV infection in HEK293T using serum from CHIKV (n = 5) and CHIKV+PbA (n = 4) groups on 15 dpi. **b**, Inhibition of CHIKV infection in HEK293T using serum from CHIKV (n = 5) and Py17x (-4 dpi)+CHIKV (n = 5) on 15 dpi. Neutralization assays were performed starting with serum dilution of 1:1000, followed by 4x serial dilutions. Data at respective dilution were compared by Mann-Whitney two-tailed analysis ($*P < 0.05$ and $**P < 0.01$). Data represent the means ± SD. Abbreviations: CHIKV, Chikungunya virus; dpi, days post infection; Py17x, *Plasmodium yoelii* 17X.

Supplementary Figure 6_Teo *et al.*, 2018

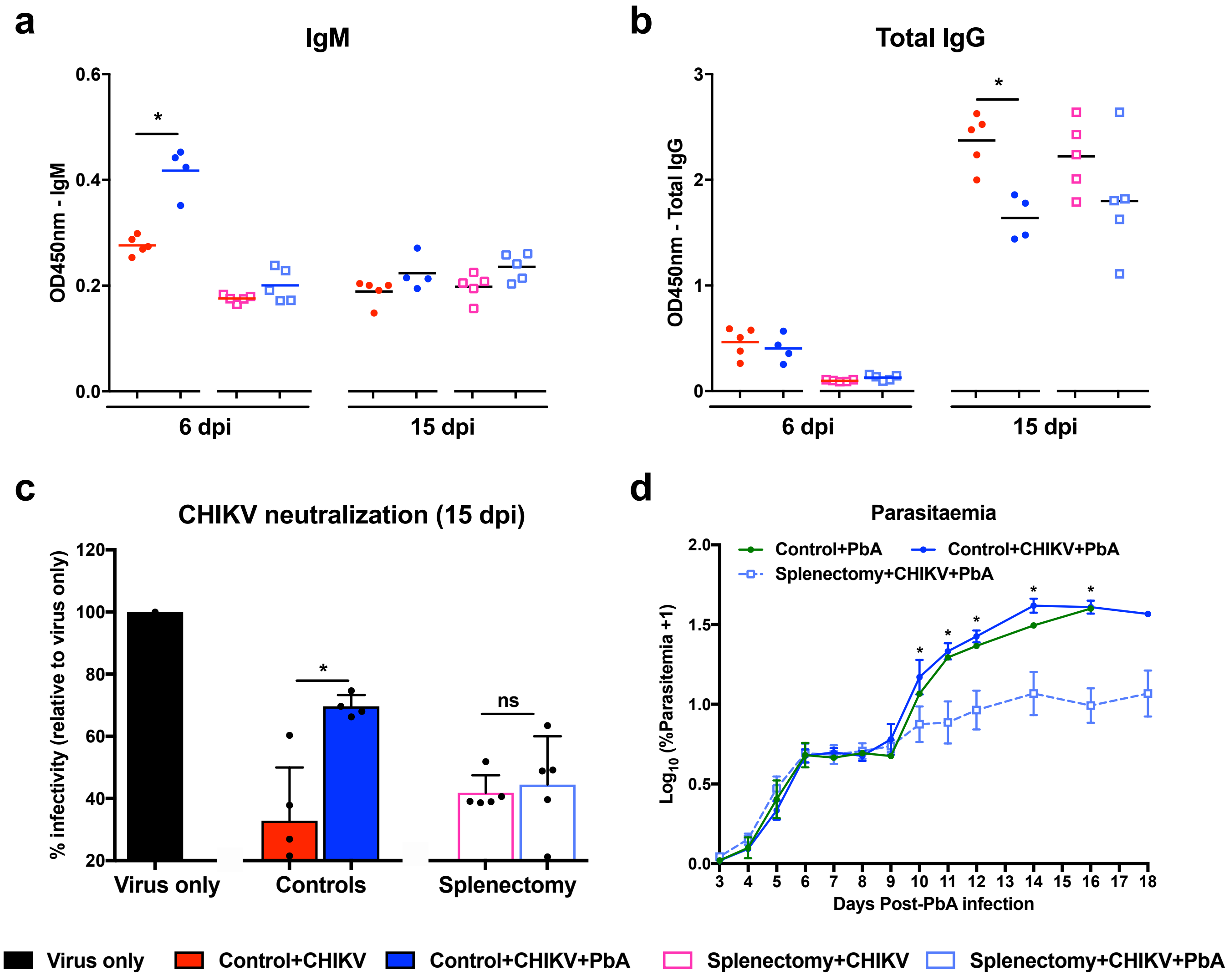

Supplementary Figure 7 I Splenectomy remove effects of co-infection induced suppression in CHIKV-specific antibodies and CHIKV neutralization, and reduce parasitaemia of co-infected mice. CHIKV-specific **a**, IgM and **b**, total IgG titer in the sera Control+CHIKV (*n* = 5), Control+CHIKV+PbA (*n* = 4), Splenectomy+CHIKV (*n* = 5) and Splenectomy+CHIKV+PbA (*n* = 5) at 6 dpi and 15 dpi. **c**, Neutralization of CHIKV infection in HEK293T cells using serum (1:250 dilutions) from Control+CHIKV (*n* = 5), Control+CHIKV+PbA (*n* = 4), Splenectomy+CHIKV (*n* = 5) and Splenectomy+CHIKV+PbA (*n* = 5) groups at 15 dpi. CHIKV-specific IgM and IgG titers were determined by virion-based ELISA at 1:250 and 1:2,000 dilution of the test serum, respectively. Data from co-infected mice were compared against their respective CHIKV infected controls by Mann-Whitney two-tailed analysis (*$P < 0.05$). **d,** Parasitaemia of Control+PbA (*n* = 5), Control+CHIKV+PbA (*n* = 4) and Splenectomy +CHIKV+PbA (*n* = 5). Data of Control+CHIKV+PbA was compared against Splenectomy+CHIKV+PbA by Mann-Whitney two-tailed analysis (*$P < 0.05$). Statistical comparison with control+PbA group could not be conducted because 80% of the mice died of ECM by 8 dpi. Data represent the means ± SD. Abbreviations: CHIKV, Chikungunya virus; ECM, experimental cerebral malaria; dpi, days post infection; ns, not significant; OD, optical density; PbA, *Plasmodium berghei*-ANKA.

Supplementary Figure 7_Teo *et al.*, 2018

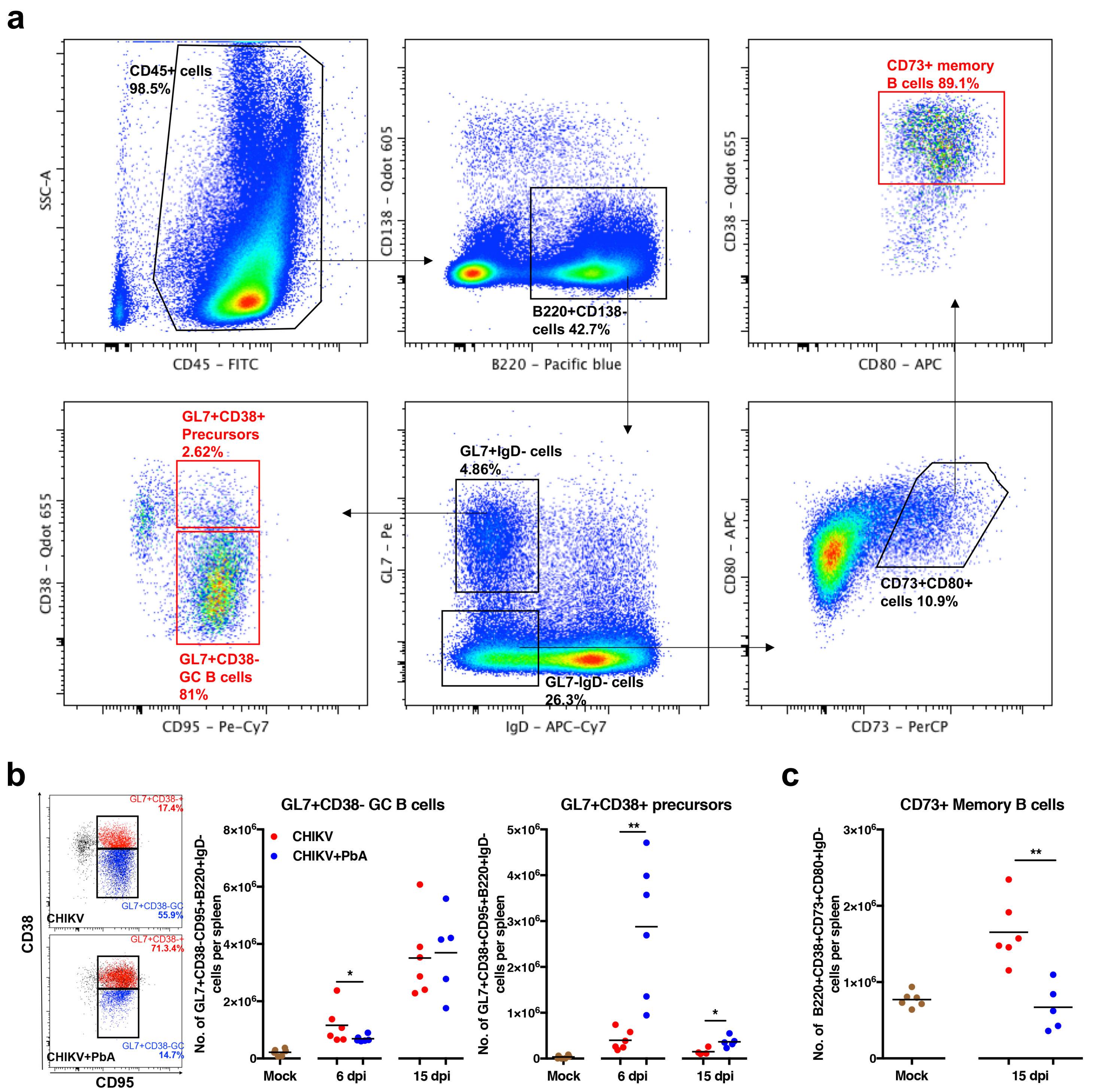

Supplementary Figure 8 I *Plasmodium* and CHIKV co-infection delays germinal center (GC) B-cell and CD73+ memory B-cell generation in the spleen. a, Gating strategy for different subsets of B cell in the spleen. Subsets that were plotted in **b** and **c** is highlighted in red. **b**, GL7+CD95+CD38- GC B cells and GL7+CD95+CD38- precursors in the spleen of CHIKV ($n \geq 5$) and CHIKV+PbA ($n \geq 5$) groups at 6 and 15 dpi. Representative dot plots displaying 3,000 events with gating on B220+GL7+IgD-, demonstrating reduction of GL7+CD95+CD38- GC B cells in co-infected mice at 6 dpi. **c**, GC-dependent CD73+ Memory B cells in the spleen of CHIKV ($n \geq 5$) and CHIKV+PbA ($n \geq 5$) groups at 15 dpi. All data were analyzed by Mann-Whitney two-tailed test (*$P < 0.05$ and **$P < 0.01$). Abbreviations: CHIKV, Chikungunya virus; GC, germinal center; PbA, *Plasmodium berghei*-ANKA.

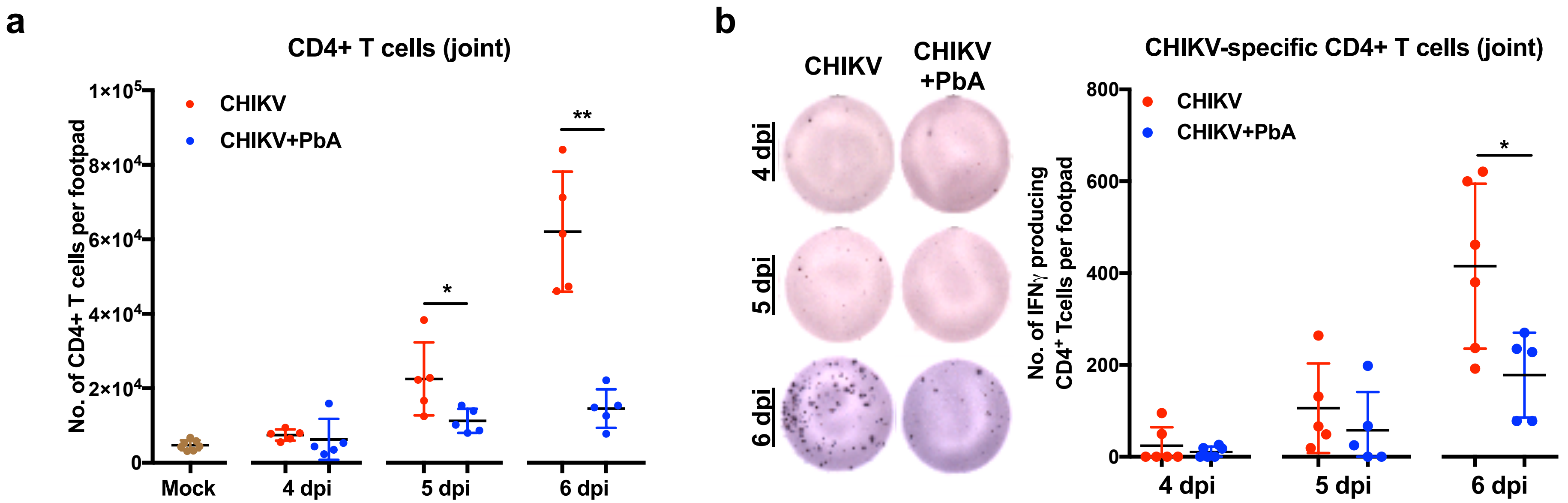

Supplementary Figure 9 I Total and CHIKV-specific CD4+ T-cell induction kinetics in the joints during CHIKV and CHIKV+PbA co-infection. Quantification of **a,** total CD4+ T cells (flow cytometry) and **b,** CHIKV-specific CD4+ T cells (ELISPOT) in the joints of CHIKV and CHIKV+PbA-infected mice ($n \geq 5$ per group) at 4, 5 and 6 dpi. **b**, Representative images of ELISPOT, displaying number of spots produced by isolated CD4+ T cells from 100,000 footpad cells. All data were analyzed by Mann-Whitney two-tailed test (*$P < 0.05$ and **$P < 0.01$). Data represent the means ± SD. Abbreviations: CHIKV, Chikungunya virus; dip, days post infection; PbA, *Plasmodium berghei*-ANKA.

Supplementary Figure 9_Teo *et al.*, 2018

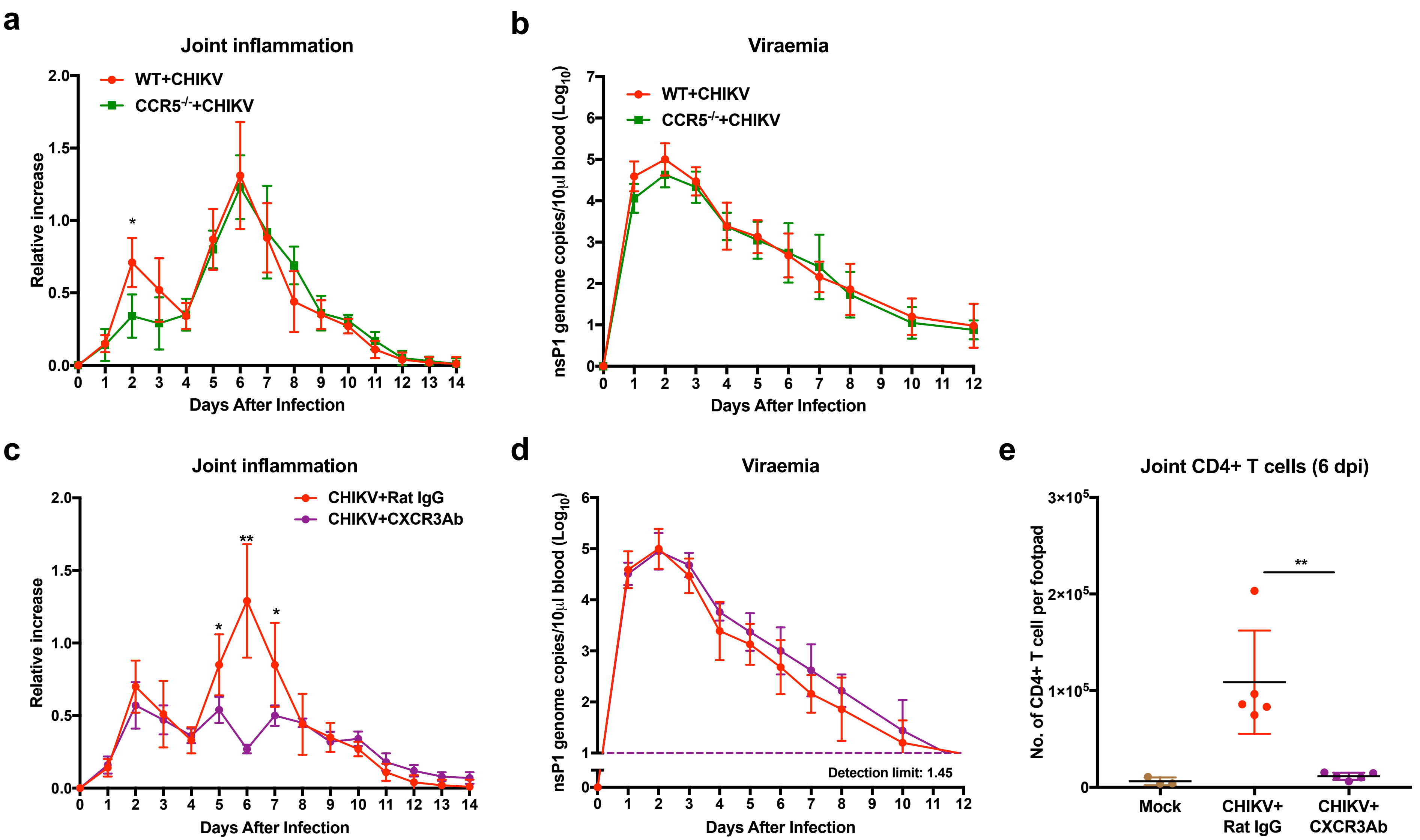

Supplementary Figure 10 I CXCR3 and not CCR5 is functionally important for CD4+ T cells mediated CHIKV inflammation. a, Joint inflammation and **b,** viraemia of WT+CHIKV (n = 5) and CCR5$^{-/-}$+CHIKV (n = 4) groups. All data were analyzed by Mann-Whitney two-tailed test (*P < 0.05). Data are representative of two independent experiments. **d**, Joint inflammation and **e,** viraemia of CHIKV+Rat IgG (n = 5) and CHIKV+CXCR3Ab (n = 5) groups. Data were analyzed by Mann-Whitney two-tailed test (*P < 0.05 and **P < 0.01). Data shown are representative of two independent experiments. **c,** Numbers of CD4+ T cells in the footpad at 6 dpi in mock (n = 3), CHIKV+Rat IgG (n = 5) and CHIKV+CXCR3Ab (n = 5) groups. Data from the CHIKV+Rat IgG group was compared with the CHIKV+CXCR3Ab group by Mann-Whitney two-tailed test (**P < 0.01). CD4+ T cells are defined as CD45+CD3+CD4+ cells. Data represent the means ± SD. Abbreviations: CHIKV, Chikungunya virus; WT, wild type.

Supplementary Figure 10_Teo *et al.*, 2018

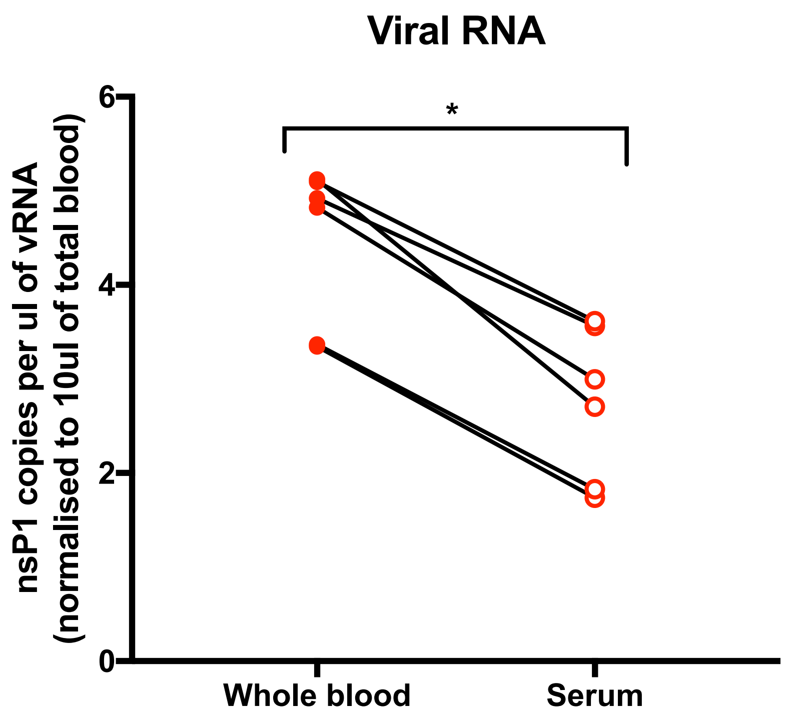

Supplementary Figure 11 I Viral RNA comparison between whole blood and serum samples. Viral RNA was quantified using paired samples from animals at 3 dpi (n = 6). For serum extraction, 30μl of blood was used to obtain 14-16μl of serum and 10μl of serum were used for viral RNA extraction and qRT-PCR. 10μl of whole blood were used for viral RNA extraction and qRT-PCR. All data were normalized to RNA copies in 10μl of total blood and compared by Wilcoxon matched-pairs signed rank test (*$P < 0.05$).

Supplementary Figure 11_Teo *et al*., 2018

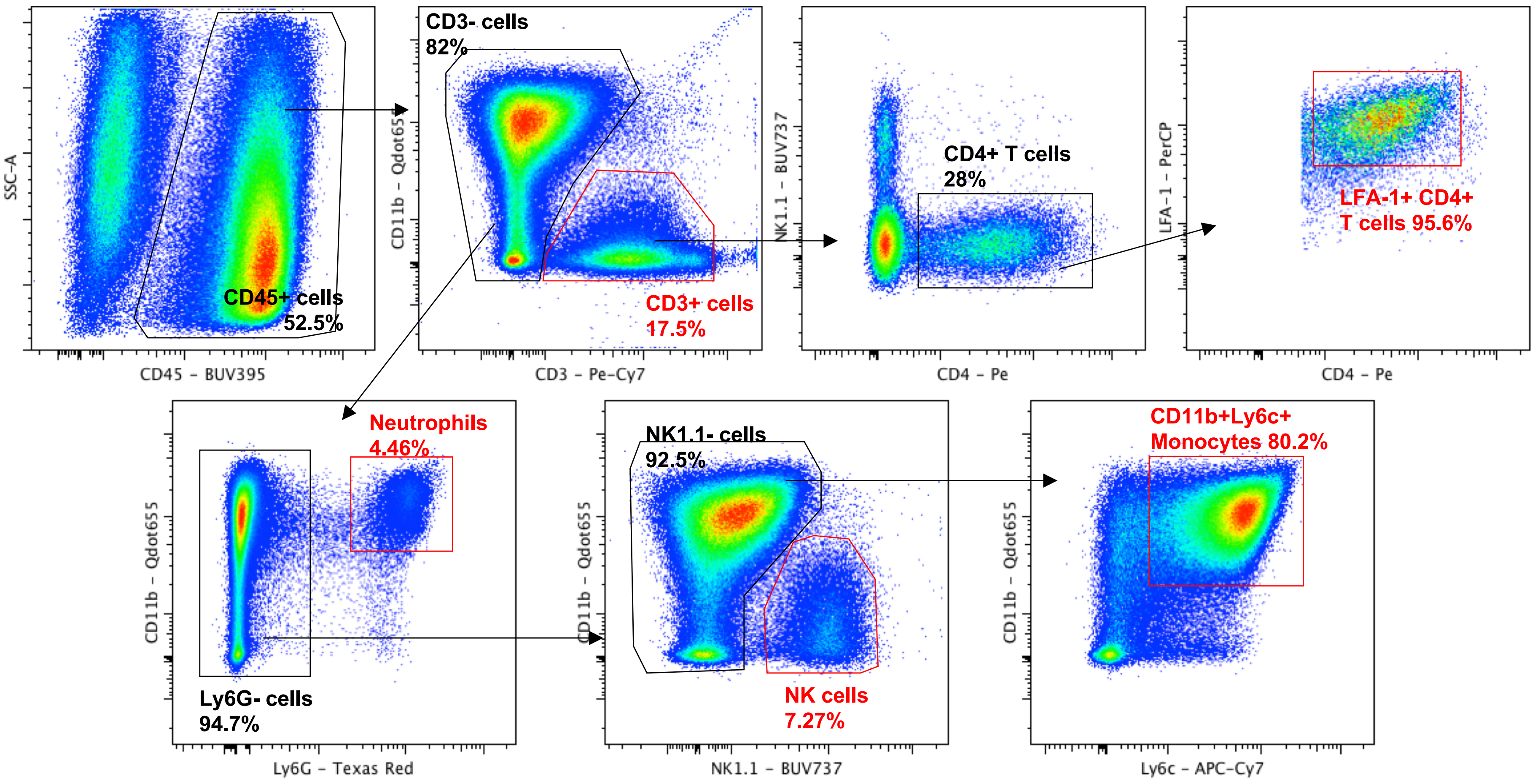

Supplementary Figure 12 I Representative gating strategy for leucocytes profiling in CHIKV-infected footpad on 6 dpi. Gating strategy used for quantification of CD3+ cells, LFA-1+ cells, neutrophils, NK cells and CD11b+Ly6c+ monocytes displayed in Figure 5c, d, f, g and h were highlighted in red.

Supplementary Figure 12_Teo *et al.*, 2018

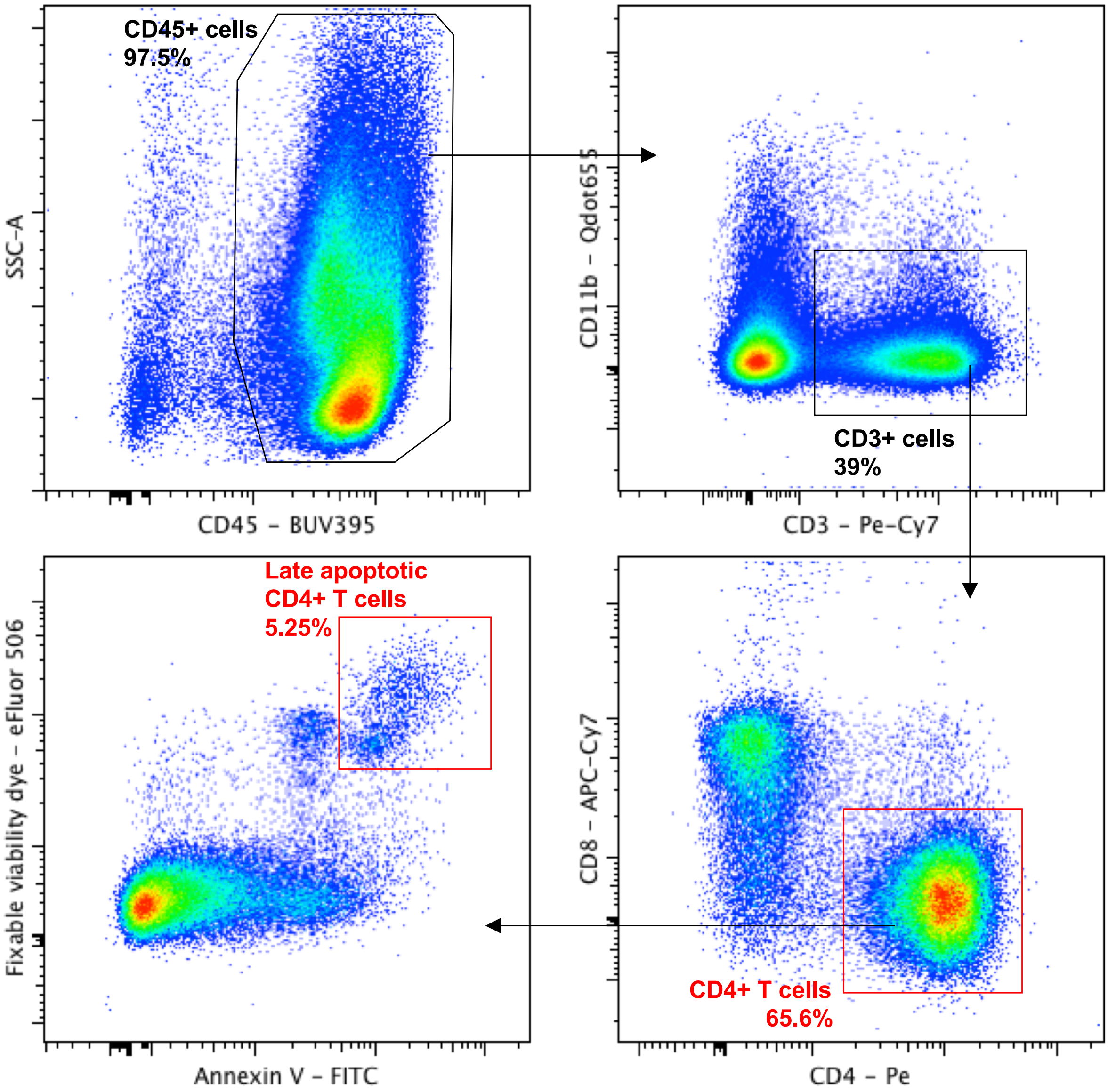

Supplementary Figure 13 I Representative gating strategy for apoptotic CD4+ T cells in the popliteal lymph nodes CHIKV-infected mice. Gating strategy used for quantification of CD4+ T cells, and late apoptotic CD4+ T cells displayed in Figure 6f and g were highlighted in red.

Supplementary Figure 13_Teo *et al*., 2018
