# Supplementary material for: Plasmodium co-infection protects against chikungunya virus-induced pathologies
Source: Nat Commun. 2018 Sep 25;9:3905. doi: 10.1038/s41467-018-06227-9 (PMC6156405; doi:10.1038/s41467-018-06227-9)
Supplement: Supplementary file 2 — Description of Additional Supplementary Files [file 41467_2018_6227_MOESM2_ESM.pdf]

Description of Additional Supplementary Files

File Name: **Supplementary Movie 1**
Description: **Kinetics of CHIKV dissemination and replication in the tissues during concurrent CHIKV+PbA co-infection.** Representative images of bioluminescence signal (CHIKV viral load) in the tissues of CHIKV-infected (*n* = 5) and CHIKV+PbA coinfected (*n* = 6) mice. All images were taken at 8 min post luciferin injection with exposure setting of 60s.

File Name: **Supplementary Movie 2**
Description: **Kinetics of CHIKV dissemination and replication in the tissues during sequential Py17x (-4 dpi)+CHIKV.** Representative images of bioluminescence signal (CHIKV viral load) in the tissues of CHIKV-infected (*n* = 7) and Py17x (-4 dpi)+CHIKV coinfected (*n* = 5) mice. All images were taken at 8 min post luciferin injection with exposure setting of 60s.

File Name: **Supplementary Movie 3**
Description: **Kinetics of CHIKV dissemination and replication in the tissues of LT$\alpha^{-/-}$ mice with concurrent CHIKV+PbA co-infection.** Representative images of bioluminescence signal (CHIKV viral load) in the tissues of LT$\alpha^{-/-}$+CHIKV (*n* = 4) and LT$\alpha^{-/-}$+CHIKV+PbA coinfected (*n* = 4) mice. All images were taken at 8 min post luciferin injection with exposure setting of 60s.
